# Mapping Human Clinical Evidence for Chikungunya Vaccines: A Scoping Review of Immunogenicity, Durability, and Safety

**DOI:** 10.3390/vaccines14070598

**Published:** 2026-07-06

**Authors:** Shan Wu, Jiachen Wu, Yiu-Wing Kam

**Affiliations:** Division of Natural and Applied Science, Duke Kunshan University, Kunshan 215316, China; shan.wu@dukekunshan.edu.cn (S.W.); jiachen.wu@dukekunshan.edu.cn (J.W.)

**Keywords:** chikungunya vaccine, vaccine platform, virus-like particle vaccine, live-attenuated vaccine, inactivated vaccine, nucleic acid vaccine, neutralizing antibody, immunogenicity, safety

## Abstract

Two chikungunya (CHIKV) vaccines have now been licensed, but the human clinical evidence base remains fragmented across vaccine platforms, populations, follow-up periods, and safety settings, complicating product-specific interpretation of durability and benefit–risk. We conducted a PRISMA-ScR–guided scoping review of human CHIKV vaccine evidence indexed in PubMed, Embase, and Web of Science from January 2000 to June 2026. After screening 890 records, we included 77 sources of evidence and mapped them at both the record level and the candidate/product level. The included evidence clustered around a limited number of vaccine programs, including TSI-GSD-218, VRC-CHKVLP059-00-VP/PXVX0317/Vimkunya, MV-CHIK/V184, VLA1553/IXCHIQ, ChAdOx1 Chik, and mRNA-1388/VAL-181388. Late-stage and post-authorization evidence was concentrated mainly in VLA1553/IXCHIQ and PXVX0317/Vimkunya, whereas viral-vector and mRNA candidates remained largely restricted to early-phase adult studies. Evidence has expanded to adolescents and adults aged ≥65 years for selected products but remains limited or product-specific for children, pregnant individuals, immunocompromised populations, and medically complex older adults. Short-term trial safety data were characterized primarily by mild or moderate local and systemic reactogenicity, while post-authorization safety evidence remains recent and concentrated in licensed products. This scoping review provides a structured evidence map for CHIKV vaccine development and highlights priorities for standardized immunogenicity assessment, longer-term durability data, broader population representation, endemic-region effectiveness studies, and continued post-marketing surveillance.

## 1. Introduction

Chikungunya virus (CHIKV) has shifted from a neglected infection to a recurring global threat. Since the mid-2000s, large outbreaks have spread across Africa, Asia, the Indian Ocean and Pacific regions, Europe, and the Americas, driven by travel, urban growth, and suitable climates [1]. Illness begins as an acute febrile syndrome but often includes severe, symmetric arthralgia; in a subset of patients, pain persists for months and can be disabling [2]. These features create different programmatic needs for travelers, high-risk workers, and communities experiencing recurrent local transmission [3]. Despite the growing geographic expansion of CHIKV, prevention options remained limited until the recent licensure of vaccines.

CHIKV is an enveloped, positive-sense RNA alphavirus (~11–12 kb). Its genome encodes four non-structural proteins (nsP1–nsP4) involved in replication and the structural proteins, including capsid (C) and the envelope glycoproteins E1/E2 (with E3 and 6K/TF) [4]. E2 helps the virus attach to host cells, while E1 drives fusion in the acidic endosome [5]. Three major lineages circulate—West African, East/Central/South African (ECSA, including the Indian Ocean lineage), and Asian—and adaptive substitutions such as E1-A226V increase fitness in *Aedes albopictus*, helping explain outbreaks in temperate settings [6]. These virologic features inform vaccine design, as most candidates present E1/E2 antigens and clinical trials commonly use neutralizing antibodies as primary immunogenicity endpoints [7].

In clinical care, after a 3–7 day incubation (range 1–12), illness starts suddenly with high fever (often ≥39 °C) and severe, symmetric joint pain, especially in small joints of the hands, wrists, and ankles [8,9,10]. Rash, myalgia, headache, and fatigue are common [8]. Lab tests often show lymphopenia and mild transaminase elevation; thrombocytopenia is usually less marked than in Dengue [11]. Fever settles in about a week, but joint pain can last weeks to months, and some patients develop chronic arthritis-like symptoms [12]. Although severe disease is uncommon, severe or atypical manifestations may occur, including neonatal infection (around birth), neurologic problems (e.g., encephalitis), and myocarditis. The risk of severe disease is higher in adults aged ≥65 years and in those with comorbidities [13]. Compared with Dengue, CHIKV tends to cause more disabling arthralgia and rash, while Dengue shows deeper thrombocytopenia and severe manifestations (including hemorrhage and plasma leakage)—still, lab confirmation is essential [14,15]. For diagnostic support, NAAT/RT-PCR is useful during the first week of illness [11]. IgM/IgG testing is helpful thereafter, where neutralization assays can provide confirmatory evidence of prior or recent CHIKV infection when available [11]. Because clinical syndromes overlap with Dengue and Zika, parallel testing is advised where these viruses co-circulate. This clinical and diagnostic context also explains why vaccine trials have relied heavily on neutralizing antibody responses as immunologic endpoints, even though validated correlates of protection remain incompletely established [11,16].

The CHIKV vaccine field has recently moved from early-phase clinical development toward licensure, immune-bridging, and early post-authorization surveillance. Two single-dose vaccines are now licensed: a live-attenuated product and a VLP/recombinant product [17]. However, the human evidence base remains difficult to interpret across products because candidates differ in platform, trial design, dose schedule, population, assay method, endpoint definition, and follow-up window. Neutralizing antibodies are used as primary immunologic endpoints, but validated correlates of protection are not yet established, as neutralization thresholds vary by assay and viral lineage [17]. Sustained durability beyond 6–12 months is rarely characterized clinically, and only a minority of programs report follow-up beyond two years. Furthermore, evidence specific to key priority populations (adults ≥ 65 years, pregnant persons, immunocompromised individuals, and pediatric cohorts) remains limited [17,18]. At the same time, the evidence base is rapidly expanding: VLA1553/IXCHIQ has been evaluated in pivotal phase 3 studies and subsequently licensed in several jurisdictions, while PXVX0317/Vimkunya has generated pivotal phase III evidence supporting its licensed-product evidence base [19,20]. Emerging evidence now includes age- and population-specific assessments, including adolescents, older adults, children, and individuals with prior CHIKV exposure, as well as post-marketing safety data for IXCHIQ [21]. Together, these developments create a need for an updated synthesis of the human clinical evidence across vaccine platforms, populations, study phases, and follow-up windows.

This scoping review focuses on human clinical evidence (interventional trials and observational or post-marketing data). This study has three primary objectives: to map immunogenicity data across vaccine platforms, populations, and time points; to summarize the durability of immune responses at commonly reported follow-up windows; and to compile safety data from clinical trials and real-world surveillance, highlighting areas of consistency and uncertainty. By mapping evidence across platforms, populations, study phases, and follow-up windows, this review aims to provide a structured overview of the current clinical evidence base and identify priorities for future vaccine evaluation and implementation.

## 2. Methods and Materials

We conducted a scoping review of human clinical evidence on CHIKV vaccines and reported it in accordance with the PRISMA Extension for Scoping Reviews (PRISMA-ScR) guidelines [22]. Before screening, the review team developed an internal protocol specifying the review question, eligibility criteria, databases, screening process, data-charting items, and synthesis approach. The objective was to identify sources of human clinical evidence describing the development, clinical evaluation, immunogenicity, durability, safety, or post-authorization assessment of vaccines developed against CHIKV. Eligible sources included interventional clinical trials, extension or follow-up analyses, age- or subgroup-specific analyses, dose/schedule or lot-to-lot consistency studies, safety analyses, post-marketing surveillance reports, and indexed protocol or registry records directly linked to a human prophylactic CHIKV vaccine development program. Sources were excluded if they were review-only records without original human vaccine evidence, focused exclusively on animal or preclinical models, were not relevant to prophylactic CHIKV vaccine development or evaluation, or could not be retrieved for full-text assessment.

The literature search was performed in PubMed, Embase, and Web of Science and covered publications from 1 January 2000 to June 2026. Searches were last updated on 19 June 2026. The start date (1 January 2000) was chosen a priori to ensure coverage of contemporary CHIKV vaccine clinical research and to avoid sparsely indexed, non-comparable early literature. The full database-specific search strategy (keywords and Boolean operators) and the completed PRISMA-ScR checklist are provided in Supplementary Materials. We did not conduct a standalone manual search of trial registries; however, indexed registry or protocol records retrieved through the bibliographic databases were eligible. Therefore, non-indexed registry records and unpublished trial results may not have been captured. Although we considered records in any language at screening, only English full texts were included for data charting due to feasibility constraints. Protocol-only or registry-only records were retained for evidence mapping and traceability but were not treated as extractable outcome evidence unless immunogenicity, durability, or safety results were available.

Two co-authors (Shan Wu and Jiachen Wu) independently screened titles and abstracts, followed by full-text assessment of potentially eligible articles. Discrepancies in study selection or data charting were resolved through discussion and consensus among the authors (Shan Wu, Jiachen Wu, and Yiu Wing Kam). Data were charted using a structured data-charting framework covering candidate/product name, platform/design, study phase or evidence category, population, age group, dose and schedule, assay type, immunogenicity endpoints, durability outcomes, safety findings, post-authorization evidence, and major evidence gaps. Because multiple records could arise from the same vaccine candidate or product, evidence was charted at both the record level and the candidate/product level. Record-level screening and traceability were retained for the PRISMA flow diagram and supplementary evidence tables, whereas Table 1 summarizes the main human clinical evidence at the candidate/product level to avoid treating multiple publications from the same vaccine program as independent candidates.

**Figure 1:**
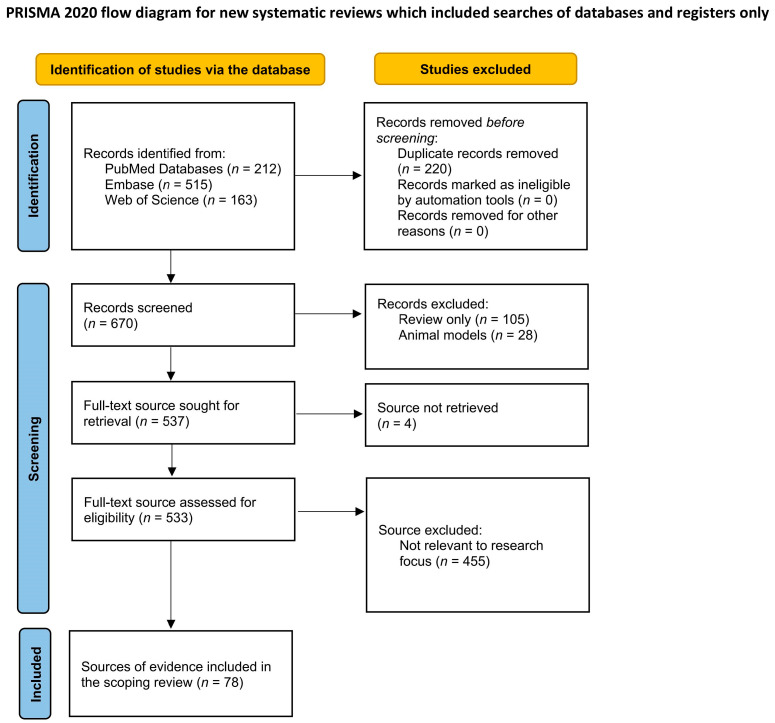
PRISMA-ScR flow diagram summarizing the identification, screening, and inclusion of sources in this scoping review of chikungunya vaccine clinical evidence. Searches were conducted in PubMed, Embase, and Web of Science from January 2000 to June 2026. After the removal of duplicates, titles and abstracts were screened for relevance to prophylactic chikungunya virus vaccines. Full texts were reviewed to confirm eligibility based on predefined criteria, including human evidence on vaccine development, safety, immunogenicity, durability, or post-authorization assessment. Sources that did not evaluate prophylactic CHIKV vaccines, lacked relevant human evidence, or did not provide extractable or mappable information were excluded. The final set of included sources was mapped at both the record level and candidate/product level.

**Figure 2:**
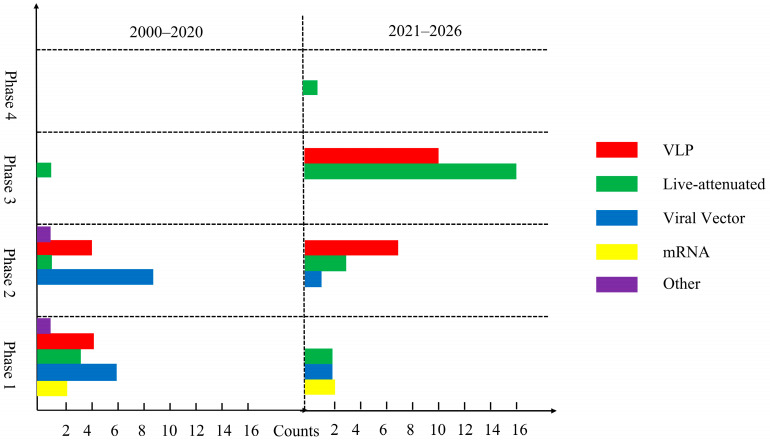
Changes in the clinical development landscape of chikungunya vaccine candidates by platform and study phase. This figure shows the distribution of included human clinical studies across vaccine platforms and clinical development phases, comparing studies published or conducted in 2000 to 2020 with those from 2021 to 2026. Overall, the recent period shows a broader and more active clinical development landscape, with studies extending across multiple platforms, including VLP-based, live-attenuated, viral-vector, and mRNA vaccines. Development phases were classified as Phase 1, Phase 2, Phase 3, Phase 4, or other/not clearly reported. The “Other” platform category includes inactivated viral vaccines, CD8 T-cell vaccine approaches, and other less common vaccine designs. CHIKV: chikungunya virus; VLP: virus-like particle.

In keeping with the scoping nature of the review, no formal assessment of study quality or risk of bias was performed. A narrative synthesis of included sources of evidence was conducted and supplemented with descriptive mapping of vaccine platform, clinical development phase, publication period, population coverage, assay type, and follow-up window. Because neutralization assays, endpoints, viral lineages, reporting units, and visit windows differed substantially across vaccine programs, no meta-analysis or pooled immunogenicity estimate was attempted. Instead, immunogenicity and durability findings were summarized descriptively within candidate/product groups and interpreted with attention to assay heterogeneity. The study selection process and results are summarized using text, figures, and tables (Figure 1 and Figure 2 and Table 1). Figure 1 summarizes study selection; Figure 2 maps evidence by platform, phase, and publication period; Table 1 presents candidate/product-level evidence; Supplementary Table S1 preserves record-level audit information; and Supplementary Table S2 summarizes extractable and contextually relevant human safety evidence.

## 3. Results

The database search identified 890 records, including 212 from PubMed, 515 from Embase, and 163 from Web of Science. After the removal of 220 duplicate records, 670 records underwent title and abstract screening. We excluded 105 review-only records and 28 animal-model records at this stage. Of 537 full-text sources sought for retrieval, four could not be retrieved, leaving 533 sources for full-text eligibility assessment. After excluding 455 sources that were not relevant to the research focus, 77 sources of evidence were included in the scoping review (Figure 1).

These 77 sources did not represent 77 independent vaccine candidates or trials. Instead, the evidence clustered around a smaller number of CHIKV vaccine programs, including TSI-GSD-218, the CHIKV VLP program (VRC-CHKVLP059-00-VP/PXVX0317/Vimkunya), MV-CHIK/V184, VLA1553/IXCHIQ, ChAdOx1 Chik, and mRNA-1388/VAL-181388. Many sources were linked to the same candidate or product and included registry or protocol records, preliminary conference abstracts, subgroup analyses, follow-up studies, safety-focused analyses, or post-marketing reports. Therefore, the synthesis is presented at two levels: Supplementary Tables S1 and S2 retain record-level transparency, whereas Table 1 summarizes the principal human clinical evidence at the candidate/product level.

### 3.1. Evidence Map, Platform Coverage, and Assay Heterogeneity

The evidence map showed uneven platform coverage. Live-attenuated and VLP/recombinant vaccine programs accounted for most late-stage and post-authorization human evidence, whereas viral-vector and mRNA candidates remained largely confined to early-phase development. Among the included candidates, VLA1553/IXCHIQ has the broadest evidence package, including phase I, pivotal phase III, lot-to-lot consistency, phase 3b persistence, adolescent phase III, pediatric phase II, and post-marketing safety evidence [19,21,38,40,41]. Within the VLP/recombinant category, early VRC VLP formulations and the later alum-hydroxide–adjuvanted PXVX0317/Vimkunya product were retained in the same candidate-level table row for readability but interpreted separately. This distinction is important because formulation, adjuvant status, schedule, assay endpoints, and follow-up windows differed across studies. The CHIKV VLP program also has phase I–III evidence, including studies in adults, adolescents aged 12–17 years, adults aged ≥65 years, prior alphavirus vaccine recipients, and serostatus subgroups [18,20]. In contrast, MV-CHIK/V184 has phase I–II adult evidence with additional cellular immunity and dose/schedule analyses, while ChAdOx1 Chik and mRNA-1388/VAL-181388 remain represented mainly by phase I adult studies [31,33,44]. TSI-GSD-218 provides an older phase II signal but has no contemporary phase III or licensure pathway in the current evidence set [23].

Assay heterogeneity was common across candidates. Immunogenicity was reported using different neutralization assays and endpoints, including PRNT_50_ or PRNT_80_, micro-PRNT_50_, NT_80_, FRNT_50_/EC_50_, IC_50_/EC_50_, and binding-antibody assays. These assays differed by virus system, endpoint definition, readout, reporting unit, viral lineage, and, where reported, cell line. For this reason, absolute GMTs are presented descriptively within candidate programs rather than pooled or ranked across platforms [7,19,20,43,44].

Within these constraints, VLA1553/IXCHIQ and CHIKV VLP candidates contributed most of the late-stage immunogenicity evidence. For VLA1553/IXCHIQ, adult phase III studies reported Day 29 micro-PRNT_50_ GMTs around 2954–3362 and Month 6 GMTs around 735–752, while persistence studies reported GMTs of approximately 785 at Year 2 and 610 at Year 4. Adolescents had Day 28 GMTs around 3856, with Month 6 and Month 12 GMTs around 1360 and 1284, respectively [18,19,20,35,39,40]. Pediatric interim data also showed early neutralizing responses after full-dose VLA1553, although longer follow-up in children remains limited [41]. For the CHIKV VLP program, phase III single-dose data reported Day 22 NT_80_ GMTs of 1618 in participants aged 12–64 years and 724 in adults aged ≥65 years, with Month 6 GMTs of 338 and 233, respectively. Phase II/adjuvanted and secondary analyses also provided evidence of durable neutralization and cross-lineage breadth, including FRNT/EC50-based responses in selected subsets [29].

### 3.2. Age, Dose, and Schedule Patterns

Age-related patterns were most clearly visible in vaccine programs that included adolescent, adult, and older-adult cohorts. In the CHIKV VLP program, the phase III single-dose study reported higher Day 22 NT_80_ GMTs in participants aged 12–64 years than in adults aged ≥65 years, with a similar direction of difference at Month 6 [18,20]. For VLA1553/IXCHIQ, adult, adolescent, and pediatric studies all reported early neutralizing-antibody responses, but the available follow-up differed by age group [19,39,41]. Adolescents had reported responses through Month 12, whereas pediatric evidence currently provides mainly early post-vaccination immunogenicity [39,41]. Older-adult evidence was most clearly represented in the dedicated PXVX0317/Vimkunya study in adults aged ≥65 years and in older-adult subgroups or analyses from VLA1553/IXCHIQ programs [18,35]. Reported GMTs differed by age group in some programs, but the included studies were not designed to establish the clinical significance of these differences.

Dose and schedule effects were also candidate-specific. Earlier VLP studies used multi-dose regimens, whereas later PXVX0317/Vimkunya pivotal trials used a single 40 μg intramuscular dose [7,20,25]. Phase II studies also evaluated booster, adjuvanted, and unadjuvanted regimens [24]. MV-CHIK/V184 studies evaluated different dose levels and intervals between the first and second vaccination, with later dosing analyses suggesting higher titers with higher doses and longer intervals [32,33]. The mRNA-1388/VAL-181388 phase I study showed a dose–response pattern after two doses, with higher GMTs in the 50 μg and 100 μg groups than in the 25 μg group [44]. For VLA1553/IXCHIQ, most later-stage studies used a single-dose schedule, while pediatric data included half-dose and full-dose comparisons [19,36,41,45]. Because dose, schedule, platform design, adjuvant use, assay method, and follow-up duration differed across candidates, these findings are summarized descriptively rather than compared quantitatively across products.

### 3.3. Study Populations and Settings

Across the evidence map, participants were still predominantly healthy adults, especially in earlier phase I–II studies conducted in high-income, non-endemic settings. However, the recent evidence base shows broader population coverage than earlier phase I–II development programs. Later-stage records now include adolescents aged 12–17 years, adults aged ≥65 years, and children aged 1–11 years, although these data remain concentrated within a limited number of products, mainly VLA1553/IXCHIQ and PXVX0317/Vimkunya [18,39,41].

For adolescents, evidence includes the VLA1553/IXCHIQ phase III program in Brazil and the PXVX0317/Vimkunya phase III program that enrolled participants aged 12–64 years [20,39]. For older adults, the most clearly dedicated evidence comes from the PXVX0317/Vimkunya phase III study in adults aged ≥65 years, while VLA1553/IXCHIQ adult studies and persistence analyses included older-adult subgroups [18,40]. Pediatric evidence has also emerged for VLA1553/IXCHIQ, including a phase II study in children aged 1–11 years in the Dominican Republic and Honduras, although pediatric evidence remains early and product-specific [41]. By contrast, ChAdOx1 Chik, mRNA-1388/VAL-181388, MV-CHIK/V184, and TSI-GSD-218 remain represented mainly by healthy adult cohorts.

Geographically, much of the clinical evidence remains concentrated in the United States and Europe, but endemic-region evidence is no longer absent. VLA1553/IXCHIQ adolescent and pediatric studies included participants from Brazil, the Dominican Republic, and Honduras [39,41]. Even so, evidence from high-burden settings in Africa, South Asia, and many parts of Southeast Asia remains limited, and most late-stage evidence continues to come from a small number of vaccine programs rather than from a geographically balanced development pipeline.

Important population gaps remain. No included trial prospectively enrolled pregnant individuals as a target population. Pregnancy exposures were reported incidentally in adult VLA1553/IXCHIQ safety data, but these observations are not sufficient to establish pregnancy-specific safety or effectiveness [37]. Immunocompromised populations also remain undercharacterized. Regulatory risk-management information for VIMKUNYA indicates that immunocompromised or immunodeficient individuals, including individuals with HIV, were not included in the clinical development program and that post-authorization pharmacovigilance will monitor potential use in immunocompromised individuals [46]. Similarly, medically complex older adults and individuals with substantial comorbidities remain insufficiently represented, particularly for live-attenuated platforms, for which dedicated evidence in immunocompromised populations remains limited.

### 3.4. Phase–Time Distribution

Figure 2 illustrates the temporal shift in the evidence base. Before 2021, included sources were mainly phase I–II studies across early live-attenuated, VLP, viral-vector, and mRNA programs. From 2021 onward, the evidence base expanded substantially, but phase III and post-authorization evidence remained concentrated in VLA1553/IXCHIQ and PXVX0317/Vimkunya. Several records in later periods represented protocols, abstracts, subgroup analyses, persistence follow-up, safety analyses, or post-marketing reports rather than independent vaccine candidates. Live-attenuated and VLP/recombinant platforms account for nearly all late-stage and post-authorization evidence, whereas MV-CHIK/V184, ChAdOx1 Chik, and mRNA-1388/VAL-181388 remain largely restricted to phase I–II adult studies [31,33,43,44,47]. Therefore, the phase-time distribution should be interpreted as evidence of activity by source type and platform, not as a count of independent products.

Consistent with this distribution, longer-term durability and post-authorization safety records were concentrated in licensed or late-stage products. Longer-term follow-up is now available for VLA1553/IXCHIQ, including persistence data to two and four years, and selected VLP studies provide follow-up through Month 6 and, in some cases, beyond the immediate post-vaccination period [35,40]. However, multi-year durability remains limited or unavailable for most viral-vector and mRNA candidates. Similarly, real-world safety evidence has begun to emerge for licensed products, especially IXCHIQ/VLA1553 [21]. Post-authorization evidence remains recent and differs from trial evidence in source population, follow-up structure, and adverse-event ascertainment [21].

### 3.5. Safety Profile Across Platforms

Safety evidence was summarized narratively because sample sizes, follow-up windows, adverse-event definitions, and ascertainment methods differed across candidates and evidence sources. Therefore, the findings should be interpreted as product-specific safety summaries rather than comparative safety estimates. Across trial reports, local reactions such as injection-site pain and systemic events such as headache, fatigue, myalgia, fever, and arthralgia were commonly reported; most reported events were mild or moderate. The amount of safety evidence varied substantially by platform. VLA1553/IXCHIQ and PXVX0317/Vimkunya provided the largest safety datasets in the included evidence map, including phase III trials and additional safety-focused analyses, whereas MV-CHIK/V184, ChAdOx1 Chik, mRNA-1388/VAL-181388, and TSI-GSD-218 were supported mainly by smaller phase I–II studies [18,19,20,21,23,27,31,39,43,47].

For VLP/recombinant vaccines, phase I–III studies generally reported mostly mild local or systemic reactogenicity [18,20]. Earlier VLP trials reported no vaccine-related serious adverse events. In the phase III PXVX0317/Vimkunya study among participants aged 12–64 years, adverse events were more frequent in vaccine recipients than placebo recipients, mainly because of local and systemic solicited events [20]. In the dedicated study of adults aged ≥65 years, overall adverse-event frequencies were similar between vaccine and placebo groups, and no treatment-related serious adverse events or deaths were reported [18]. Follow-up in these phase III studies was mainly through six months [18,20].

For VLA1553/IXCHIQ, reactogenicity was more frequently reported in vaccine recipients than placebo recipients in adult trial datasets [19]. Common systemic events included headache, fatigue, myalgia, arthralgia, and influenza-like symptoms, while arthralgia was reported more frequently after VLA1553/IXCHIQ than after placebo [19]. Serious adverse events were uncommon in clinical trials. A small number of events were assessed as vaccine-related in adult trial datasets, and reported related serious adverse events resolved without sequelae [19]. In adolescent and pediatric studies, solicited systemic and local events were also reported, with adolescent data showing more related adverse events after VLA1553/IXCHIQ than placebo and pediatric interim data showing mainly fever, headache, tenderness, and injection-site pain [19]. Early post-authorization surveillance for IXCHIQ/VLA1553 also reported serious adverse events during real-world use. In the early post-authorization safety review, serious adverse-event reports were concentrated among older adults and individuals with comorbidities, including systemic chikungunya-like, neurological, cardiac, and renal events [21]. Because these data came from surveillance systems rather than randomized trials, they are reported here as post-authorization safety signals rather than as directly comparable incidence estimates.

For earlier-stage candidates, available safety data were more limited. TSI-GSD-218, MV-CHIK/V184, ChAdOx1 Chik, and mRNA-1388/VAL-181388 were mainly supported by small adult phase I or phase II studies, with follow-up windows and adverse-event definitions varying across studies. These studies generally reported mostly mild or moderate local and systemic adverse events, but their small sample sizes limit interpretation of rare adverse events, long-term safety, and safety in underrepresented populations.

## 4. Discussion

This scoping review maps the current human evidence for CHIKV vaccines and shows rapid late-stage progress concentrated in live-attenuated and VLP/recombinant platforms, while viral-vectored and mRNA candidates remain early. Cross-study heterogeneity, limited follow-up, and under-representation of priority groups and endemic settings constrain interpretation and public-health relevance. These findings have three main implications: vaccine evidence should be interpreted at the product level rather than the platform level alone. Immunogenicity results require careful attention to assay and endpoint differences, and future evaluation should prioritize durability, population representativeness, endemic-region effectiveness, and post-authorization safety surveillance.

### 4.1. Immunogenicity and Assay Limitations

The human clinical evidence base for CHIKV vaccines has expanded substantially in recent years, with later-stage evidence now concentrated in live-attenuated VLA1553/IXCHIQ and VLP/recombinant PXVX0317/Vimkunya programs. Licensed products have moved the field toward immune-bridging and post-authorization evaluation, although assay heterogeneity, the absence of validated correlates, and limited multi-year follow-up for most non-live platforms continue to constrain inference.

Despite these advances, important uncertainties remain. Evidence regarding long-term protection is still concentrated in a small number of vaccine programs, and direct comparisons across platforms continue to be limited by assay heterogeneity and differences in study design [7,18,19,44]. Furthermore, post-marketing experience remains relatively recent, and continued pharmacovigilance will be essential to clarify rare adverse events and long-term benefit-risk profiles across different recipient populations [21].

Interpretation of these findings is constrained by marked heterogeneity in serologic assays. Across studies, neutralization platforms varied widely (PRNT, μPRNT, FRNT, and NT_50_/NT_80_), assays used different CHIKV lineages, and results were reported in non-interchangeable units [7,18,19,20,44]. These assays differ in virus system, readout, endpoint definition, and reporting scale. Therefore, absolute GMTs should not be interpreted as interchangeable measures across studies or platforms. The limitation is particularly important when comparing live-attenuated, VLP/recombinant, viral-vectored, and mRNA-based approaches, because apparent differences in GMT magnitude may partly reflect assay design rather than only biological differences in vaccine-induced immunity.

At the same time, assay heterogeneity should not obscure several biologically meaningful patterns. Within individual vaccine programs, neutralizing-antibody responses provide useful evidence of immune activation, dose response, boosting effects, and durability when the same assay is used across time points. In addition, several studies have begun to address the breadth of vaccine-induced responses rather than only their magnitude [7,19,39,40,42]. VLP-based vaccination has been associated with broadly neutralizing antibody responses against diverse alphaviruses, while ChAdOx1 Chik induced neutralizing antibodies against four CHIKV lineages after a single dose in a phase 1 trial [29,43]. These findings suggest that cross-lineage breadth is an important dimension of vaccine evaluation, especially because CHIKV lineages differ geographically and outbreak settings may involve heterogeneous circulating strains [43].

Neutralizing antibodies also represent only one component of vaccine-induced immunity. Although they remain the dominant immunogenicity endpoint in CHIKV vaccine trials, cellular and non-neutralizing antibody responses may contribute to durability, breadth, and recall responses, particularly for live-vectored platforms. Evidence from MV-CHIK studies showing CHIKV-specific CD4+ T-cell responses supports the need to interpret immunogenicity more broadly than neutralizing GMTs alone [33]. However, cellular immunity, binding antibody profiles, and functional non-neutralizing responses were not measured consistently across programs, limiting their integration into a comparative evidence map. Future studies should therefore standardize not only neutralization assays and sampling windows, but also reporting of cellular immunity, cross-lineage neutralization, and candidate correlates of protection.

### 4.2. Study Design, Population Representation, and Post-Licensure Needs

Study design and population coverage also shape the strength of the current evidence base. Although Figure 2 demonstrates a clear expansion of late-phase clinical development since 2021, Table 1 shows that this progress is concentrated within a limited number of vaccine programs, particularly VLA1553 and PXVX0317. Consequently, the apparent maturity of the field should not be interpreted as uniform across all vaccine platforms. Viral-vectored and mRNA candidates remain represented largely by early-phase studies, whereas most long-term immunogenicity, durability, and safety data originate from a small number of live-attenuated and VLP/recombinant programs. This concentration of evidence affects the certainty with which findings can be generalized across platforms and product types. Pregnant individuals, young children, older adults with comorbidities, and immunocompromised populations remain largely unrepresented [41]. Several late-stage studies were conducted in the United States or Europe and may not fully represent the demographic, epidemiologic, and health-system contexts of high-burden regions in Latin America, Africa, South Asia, and Southeast Asia [24,39].

Population representation has improved over time, but important gaps remain. Figure 2 illustrates that most published studies continue to focus on healthy adults, while Table 1 shows that dedicated evaluations in children, adolescents, and older adults remain relatively uncommon. Recent studies have begun to expand the evidence base beyond the populations that dominated earlier trials, including adolescents in endemic regions, children aged 1–11 years, and adults aged ≥65 years [18,41]. These developments are important because they move vaccine evaluation closer to populations relevant for outbreak control and routine implementation. However, such evidence remains concentrated within a limited number of products, and dedicated data for pregnant individuals, immunocompromised populations, and medically complex older adults remain scarce. Therefore, the external validity of many current findings remains constrained by the characteristics of enrolled trial populations.

Another important shift in the evidence landscape is the emergence of post-licensure data. As shown in Figure 2, the rapid expansion of phase 3 studies after 2021 has been accompanied by the emergence of real-world safety evidence. Clinical trials are generally designed to detect common reactogenicity events and assess short-term safety, but they are rarely powered to identify rare adverse events or characterize benefit–risk profiles in heterogeneous populations [21]. Consequently, post-marketing surveillance has become an essential complement to clinical development rather than merely a regulatory requirement. Recent post-authorization reports have provided valuable information regarding vaccine safety in older adults and other populations that were relatively underrepresented in pre-licensure studies [21]. Although such observations should not be interpreted as direct comparisons between vaccine products because of differences in surveillance systems, recipient characteristics, and reporting practices, they illustrate how pharmacovigilance can refine benefit–risk assessments beyond what is possible within clinical trials alone.

Collectively, Table 1 and Figure 2 depict a field that is transitioning from proof-of-concept and early immunogenicity studies toward implementation-relevant evidence. Nevertheless, the distribution of evidence remains uneven across platforms, populations, and geographic settings. Future research priorities therefore extend beyond demonstrating immunogenicity alone and should focus on generating evidence that is directly relevant to policy decisions, including long-term durability, effectiveness in endemic regions, inclusion of underrepresented populations, and active post-marketing surveillance. Addressing these gaps will be critical for translating promising vaccine performance into context-specific vaccination recommendations and sustainable public-health strategies.

### 4.3. Relationship to Existing Reviews and Added Value

Earlier narrative reviews described the chikungunya vaccine pipeline and highlighted the biological rationale for E1/E2-based vaccines, but they pre-dated licensure and could not evaluate longer-term clinical performance [48,49]. More recent systematic and narrative reviews concluded that major candidates achieve very high short-term seroresponse rates (often ≥95%) and acceptable short-term safety, but they were less focused on mapping how assay heterogeneity, follow-up windows, and population coverage differed across products [17,50]. Living systematic-review initiatives increasingly address safety and selected populations, but they are not primarily designed to map durability, assay heterogeneity, or cross-lineage performance across vaccine platforms.

This scoping review adds to the existing literature in several ways. Earlier reviews primarily focused on immunogenicity and short-term safety, whereas the current evidence base has expanded substantially following vaccine licensure [21,35,40]. As illustrated by Table 1 and Figure 2, recent studies have broadened evaluation beyond healthy adults to include adolescents, children, older adults, and populations from endemic settings. At the same time, post-authorization reports and longer-term follow-up studies have begun to provide evidence that was unavailable to earlier reviews [21,40]. Together, these developments reveal a field transitioning from proof-of-concept vaccine development toward implementation-relevant evaluation.

Post-licensure experience to date suggests that early safety signals may differ by platform and context: in early post-authorization reports from the United States and Germany, no serious adverse events were reported for a CHIKV VLP vaccine, including among recipients aged ≥65 years [51]. Early post-authorization reports suggest that safety observations may vary across products and recipient populations, although such findings must be interpreted cautiously because surveillance intensity, reporting systems, and baseline risks differ substantially across settings [52]. Rather than replacing evidence from randomized trials, post-marketing surveillance provides complementary information that is essential for understanding vaccine performance in populations that remain underrepresented in clinical development programs [21]. By placing these observations alongside trial findings, our review highlights the growing importance of pharmacovigilance as part of the overall CHIKV vaccine evidence ecosystem.

### 4.4. Strengths and Limitations of This Scoping Review

This scoping review has several strengths. We conducted a comprehensive search across PubMed, Embase, and Web of Science, which enabled broad coverage of the published clinical literature and reduced the likelihood that relevant studies would be missed because of database-specific indexing practices. By restricting inclusion to human clinical evidence, we were able to provide a focused synthesis that is directly relevant to vaccine evaluation, regulatory decision-making, and public-health implementation. Moreover, we explicitly place emerging post-marketing information alongside trial data to aid interpretation of benefit–risk, reflecting broader debates about how clinical trials, non-clinical studies, immunologic surrogates, and pharmacovigilance each address different dimensions of vaccine evaluation.

Several limitations should be noted. Consistent with the objectives of a scoping review, we aimed to map and synthesize the available evidence rather than formally assess risk of bias or conduct quantitative meta-analysis. Consequently, the review is not intended to provide pooled estimates of vaccine efficacy, immunogenicity, or safety. Interpretation of immunogenicity findings is further complicated by substantial heterogeneity in assay methodologies, including differences in neutralization platforms, endpoints, reporting metrics, and follow-up schedules [7,19,20,44]. These methodological differences limit direct comparisons across studies and vaccine platforms. In addition, although the evidence base has expanded considerably, much of the available data remain concentrated within a limited number of vaccine candidates, particularly VLA1553 and PXVX0317, whereas viral-vectored and mRNA platforms continue to be represented primarily by early-phase studies [33,43]. Because several retained records were registry entries, protocols, or preliminary conference abstracts, their contribution was primarily to evidence mapping and trial-traceability rather than outcome synthesis. Moreover, post-licensure evidence remains relatively recent, and ongoing pharmacovigilance will be necessary to fully characterize rare adverse events, long-term safety, and vaccine performance in populations that have historically been underrepresented in clinical trials [21]. We also did not conduct a standalone registry search, and only indexed registry/protocol records retrieved through bibliographic databases were eligible. Therefore, non-indexed registry records and unpublished results may have been missed. Finally, although records in any language were considered during screening, only English full texts were included for data charting because of feasibility constraints. Relevant non-English evidence may therefore have been missed, introducing potential language bias.

### 4.5. Implications for Policy and Research

Our findings suggest that chikungunya vaccines should not be assessed simply as ready or not ready for broad use, but rather in relation to specific products, populations, and settings. The current evidence base provides several considerations for policy deliberation, although it should not be interpreted as a substitute for effectiveness studies or formal health-economic evaluations. As summarized in Table 1 and Figure 2, the CHIKV vaccine field has progressed beyond proof-of-concept studies and now includes licensed products, phase 3 trials, and emerging post-authorization evidence. These developments suggest that the current evidence base is becoming increasingly relevant to outbreak preparedness and response planning, particularly in regions where CHIKV transmission is recurrent or expanding [19,35].

At the same time, the available evidence indicates that vaccination policies are unlikely to be universally applicable across all populations and settings. Differences in age eligibility, platform characteristics, durability data, and post-marketing safety observations highlight the importance of context-specific implementation. Recent studies in adolescents, children, and older adults have expanded the evidence base, but population-specific data remain uneven across products [18,39,41]. Therefore, decisions regarding target populations, timing of vaccination, and integration into existing immunization programs should continue to be informed by local epidemiology, population risk profiles, regulatory recommendations, and emerging pharmacovigilance data.

From a research perspective, the findings of this review suggest that future priorities extend beyond demonstrating short-term immunogenicity alone. A recurring challenge across the current literature is the substantial heterogeneity in assay platforms, neutralization endpoints, and reporting practices, which continues to complicate cross-study and cross-platform comparisons [7,19,20,43]. Greater harmonization of immunological assessment, together with the development of validated correlates of protection, would substantially improve the interpretability and comparability of future vaccine studies.

At the same time, the evolving evidence base highlights the importance of expanding evaluation into populations that remain underrepresented in clinical development. Although recent studies have broadened participation to include children, adolescents, and older adults, important gaps persist for pregnant individuals, immunocompromised populations, and medically complex older adults [18,39,41]. Further evidence from endemic regions will also be critical for understanding vaccine performance under epidemiological conditions that differ from those represented in many early clinical trials.

The recent emergence of longer-term follow-up studies and post-authorization data further illustrates how the field is moving beyond initial proof-of-concept assessments. Encouraging evidence regarding the persistence of vaccine-induced immunity is beginning to accumulate, particularly for leading vaccine candidates, yet durability data remain uneven across platforms and follow-up periods [19,40]. Similarly, expanding vaccine use will require continued active surveillance to better characterize rare adverse events and refine benefit–risk assessments in real-world settings [21].

## 5. Conclusions

This scoping review shows that chikungunya vaccine development has progressed to the point where VLP/recombinant and live-attenuated candidates generate robust early neutralizing-antibody responses and substantial late-stage human clinical evidence for selected products. Yet the clinical evidence base remains uneven across platforms, populations, geographic settings, and follow-up periods, depends on heterogeneous neutralization assays without validated correlates of protection, and includes limited comparable long-term durability data across most candidates. Although evidence has expanded to adolescents, children aged 1–11 years, and adults aged ≥65 years for selected products, pregnant individuals, immunocompromised populations, medically complex older adults, and many endemic-region populations remain insufficiently characterized.

Taken together, these findings indicate that the current evidence base is increasingly relevant to outbreak preparedness and targeted prevention planning but does not support uniform assumptions about durability, effectiveness, or safety across all products, age groups, and risk populations. Future CHIKV vaccine evaluation should prioritize standardized immunogenicity assessment, longer-term durability follow-up, broader inclusion of underrepresented populations, effectiveness studies in endemic settings, and active post-authorization surveillance, especially for rare adverse events and high-risk groups.

## Figures and Tables

**Table 1:** Candidate-level summary of human clinical evidence for chikungunya vaccine platforms.

Candidate/Product	Platform/Design	Evidence Coverage	Dose/Schedule Represented	Main Assay/Endpoint	Key Immunogenicity/Durability Findings		Main Evidence Gaps	Ref.
TSI-GSD-218	Live attenuated, serially passaged plaque-purified CHIKV vaccine derived from Thai strain 15561/CHIK 181/Clone 25.	Phase II, randomized placebo-controlled study; 73 healthy adults in the USA.		Single 0.5 mL subcutaneous dose; approx. 10^5^ pfu/dose.	PRNT50; seroconversion and antibody persistence to 1 year.	Day 28 GMT 582; 57/58 vaccinees seroconverted. Day 360 GMT 105; 85% remained seropositive at 1 year.	Small older study; healthy adults only; no contemporary phase III or licensure pathway in the current evidence set.	[23]
VLP/recombinant vaccines: early VRC VLP formulations and PXVX0317/Vimkunya	Virus-like particle vaccine presenting CHIKV structural proteins; later PXVX0317/Vimkunya formulations include aluminum hydroxide-adjuvanted regimens.	Phase I-III evidence, including adults, adolescents 12–17 years, adults ≥ 65 years, prior alphavirus vaccine recipients, and serostatus subgroup analyses.		Early VRC regimen: 3 IM doses. Endemic-region VLP trial: 20 mcg × 2, 28 days apart. PXVX0317/Vimkunya: single 40 mcg IM in pivotal trials; phase II studies also evaluated 2-dose, accelerated, booster, and adjuvanted/unadjuvanted regimens.	Neutralization assays varied across studies: IC_50_/EC_50_, NT_80_, PRNT_80_, FRNT_50_/EC_50_.	Phase III single-dose results: Day 22 NT_80_ GMT 1618 in ages 12–64 and 724 in adults ≥ 65; Month 6 GMT 338 and 233, respectively. Phase II/adjuvanted studies show durable neutralization and cross-lineage/breadth signals, including day 57 FRNT EC_50_ about 19,000 and day 182 about 1000 in a B-cell analysis subset.	Assay heterogeneity limits cross-study comparison. Pediatric data < 12 years and pregnancy/immunocompromised data remain limited in the current human evidence set.	[7,18,20,24,25,26,27,28,29]
MV-CHIK/V184	Live recombinant measles-virus vector expressing CHIKV structural proteins.	Phase I and Phase II trials in healthy adults; additional cellular immunity and dose/schedule analyses.		IM MV-CHIK; phase I evaluated 1.5 × 10^4^, 7.5 × 10^4^, and 3.0 × 10^5^ TCID_50_ with booster on day 28 or day 90. Later study evaluated 5 × 10^4^ vs. 5 × 10^5^ TCID_50_ with second dose on day 29, 85, or 169.	PRNT_50_ for neutralizing antibodies; ELISA in some studies; cellular analyses used PBMC peptide stimulation/AIM flow cytometry/ELISpot.	Phase II showed neutralizing responses after one or two immunizations; seroconversion varied by dose/schedule. Cellular substudy detected CHIKV-specific CD4+ T-cell responses in 10/12 after two vaccinations. Later Phase I dosing study found higher titers with 5 × 10^5^ TCID_50_ and longer intervals.	No phase III efficacy/immunobridging program in the current evidence set; limited long-term durability and limited priority-population data.	[30,31,32,33,34]
VLA1553/IXCHIQ	Single-dose live attenuated CHIKV vaccine based on La Reunion LR2006-OPY1/ECSA lineage with nsP3 deletion; unadjuvanted.	Phase I, pivotal Phase III, lot-to-lot consistency, Phase 3b persistence, adolescent Phase III, pediatric Phase II, and post-marketing safety evidence.		Single IM dose. Phase III adult studies used approx. 1 × 10^4^ TCID_50_ per 0.5 mL; pediatric study compared half-dose and full-dose VLA1553.	Validated micro-PRNT_50_; seroresponse/seroprotection threshold ≥ 150.	Adults: Day 29 GMT 3362 and Month 6 GMT 752 in pivotal Phase III; pooled Phase III Day 29 GMT 2954 and Month 6 GMT 735. Persistence studies show Year 2 GMT about 785 and Year 4 GMT about 610. Adolescents: Day 28 GMT 3856, Month 6 GMT 1360, Month 12 GMT 1284. Children: full-dose pooled Day 29 GMT about 2844.	Evidence base is strongest among current candidates, but pregnancy, immunocompromised populations, and real-world effectiveness remain undercharacterized; live-attenuated platform requires careful safety interpretation.	[19,21,35,36,37,38,39,40,41,42]
ChAdOx1 Chik	Replication-deficient simian adenoviral vector encoding the CHIKV full-length structural polyprotein.	Phase I first-in-human dose-escalation trial; 24 healthy adults in the UK.		Single IM dose at 5 × 10^9^, 2.5 × 10^10^, or 5 × 10^10^ viral particles.	PRNT_50_ against multiple CHIKV lineages; binding antibody and T-cell endpoints also reported.	Single dose induced IgG and T-cell responses and 100% PRNT_50_ seroconversion; neutralizing antibodies detected against four CHIKV lineages as early as 2 weeks after vaccination and followed to 6 months.	Small early-phase non-endemic adult trial; no late-stage, pediatric, older-adult, or pregnancy data in the current evidence set.	[43]
mRNA-1388/VAL-181388	mRNA vaccine candidate; lipid nanoparticle carrier; no classical adjuvant.	Phase I randomized dose-ranging trial; 60 healthy adults in a CHIKV-nonendemic region of the USA.	Two IM doses 28 days apart: 25, 50, or 100 mcg, or placebo.	PRNT_50_ EC_50_ neutralization assay against West African CHIKV strain 37997; binding antibody ELISA.	Day 56 GMTs after dose 2: 6.2 (25 mcg), 53.8 (50 mcg), and 92.8 (100 mcg). Responses persisted up to 1 year after vaccination and remained above placebo in the two higher-dose groups, although exact later GMTs were not extractable from main text/figures.		Only early-phase adult data; relatively small sample; no phase II/III, adolescent, older-adult, pediatric, pregnancy, or endemic-setting evidence in current set.	[44]

Abbreviations: CHIKV, chikungunya virus; GMT, geometric mean titer/titre; IM, intramuscular; PRNT_50_/micro-PRNT_50_, 50% plaque/micro-plaque reduction neutralization test; TCID_50_, 50% tissue culture infectious dose; VLP, virus-like particle. Registry/protocol entries without reported outcomes, superseded conference abstracts, safety-only analyses, and non-active-vaccine interventions are retained in Supplementary Table S1 for traceability. For VLP/recombinant vaccines, early VRC VLP formulations and the later alum-hydroxide–adjuvanted PXVX0317/Vimkunya product are retained within one platform row for readability, but immunogenicity and durability findings are interpreted separately by formulation/product. Absolute GMTs should not be treated as interchangeable across VLP studies because formulation, adjuvant status, dose schedule, assay endpoint, and follow-up window differ.

## Data Availability

No original participant-level data were generated in this study. The data charted and analyzed in this scoping review were derived from publicly available published articles, registry/protocol records, and post-authorization reports cited in the manuscript and summarized in the Supplementary Materials.

## Supplementary Materials

The following supporting information can be downloaded at: https://www.mdpi.com/article/10.3390/vaccines14070598/s1, Table S1: Record-level audit and extraction summary of CHIKV vaccine-related records, grouped by candidate/product; Table S2: Safety evidence summary of chikungunya vaccine candidates.
