# Supplementary material for: Mapping Human Clinical Evidence for Chikungunya Vaccines: A Scoping Review of Immunogenicity, Durability, and Safety
Source: Vaccines (Basel). 2026 Jul 6;14(7):598. doi: 10.3390/vaccines14070598 (PMC13417312; doi:10.3390/vaccines14070598)
Supplement: Supplementary file 1 [file vaccines-14-00598-s001.zip › CHIKV_Supplementary_Table_S1_sw629.pdf]

Supplementary Table S1. Record-level audit and data-charting summary of CHIKV vaccine-related records, grouped by candidate/product.

Note. Records are grouped according to the candidate/product categories used in the candidate-level main Table 1. All 77 retained records in the updated evidence library are retained for traceability. Registry/protocol records without results, preliminary abstracts superseded by full publications, safety-only analyses, non-active-vaccine interventions, and other records outside active vaccine-candidate evidence are flagged rather than expanded. When a preliminary abstract or preprint was superseded by a full publication, the full publication was prioritized for synthesis; earlier records were retained only for traceability or unique contextual information.

Abbreviations: FU = follow-up; GMT = geometric mean titer; IM = intramuscular; mAb = monoclonal antibody; NR = not reported; PRNT50/micro-PRNT50 = 50% plaque/micro-plaque reduction neutralization test; TCID50 = 50% tissue-culture infectious dose; VLP = virus-like particle.

Supplementary Table S1A. Audit classification of all 77 retained sources, grouped by candidate/product.

TSI-GSD-218 (n=1)

Candidate/product	Platform	Record status	Study context	Handling / extractable data	Ref.
TSI-GSD-218	Live attenuated CHIKV vaccine	Published clinical trial report	Phase II; n=73 adults; age 18-40 years; USA; single 0.5 mL SC dose (~10^5 pfu) or placebo; follow-up 1 year.	Extractable outcomes; used for evidence synthesis.	[1]

CHIKV VLP program (VRC-CHKVLP059; PXVX0317/Vimkunya) (n=25)

Candidate/product	Platform	Record status	Study context	Handling / extractable data	Ref.
VRC-CHKVLP059 / VRC 311	CHIKV VLP vaccine	Registry/protocol	Phase I; planned n=25; healthy adults age 18-50 years; USA/NIH; IM injections at weeks 0, 4, and 20; dose escalation 10, 20, and 40 mcg; follow-up 44 weeks.	Protocol/registry; no extractable outcomes.	[2]
VRC-CHKVLP059 / VRC 311	CHIKV VLP vaccine	Conference abstract / early report	Phase I abstract; n=25 healthy adults; IM doses on days 0, 28, and 140; dose groups 10, 20, and 40 mcg; short-term follow-up reported.	Partial; preliminary or superseded by full publication.	[3]
VRC-CHKVLP059 / VRC 311	CHIKV VLP vaccine	Published clinical trial report	Phase I; n=25; age 18-50 years; USA; IM doses at weeks 0, 4, and 20; dose groups 10, 20, or 40 mcg; follow-up 44 weeks.	Extractable outcomes; used for evidence synthesis.	[4]
VRC-CHKVLP059 / VRC 311	CHIKV VLP vaccine	Registry/protocol	Phase II registry; healthy adults age 18-60 years; endemic-region study; two IM doses on day 0 and day 28; 20 mcg CHIKV VLP or placebo; planned duration about 72 weeks.	Protocol/registry; no extractable outcomes.	[5]
CHIKV VLP vaccine	CHIKV VLP vaccine	Conference abstract / early report	Cross-genotype neutralization analysis using sera from 12 VLP vaccine recipients; reported broad neutralization against CHIKV genotypes.	Partial; limited details, retained for context.	[6]
PXVX0317 / Vimkunya	CHIKV VLP vaccine	Registry/protocol	Phase II registry; PXVX0317 formulation/schedule trial; unadjuvanted or Alhydrogel-adjuvanted regimens; schedules day 1/15, day 1/29, or day 29 only.	Protocol/registry; no extractable outcomes.	[7]
PXVX0317 / Vimkunya	CHIKV VLP vaccine	Registry/protocol	Phase II registry; healthy adults age 18-65 years; prior alphavirus vaccine recipients compared with alphavirus-naive controls; alum-adjuvanted PXVX0317.	Protocol/registry; no extractable outcomes.	[8]
VRC-CHKVLP059 / CHIKV VLP	CHIKV VLP vaccine	Published clinical trial report	Phase II; n=400 randomized; age 18-60 years; Caribbean endemic sites; two IM doses 28 days apart; 20 mcg CHIKV VLP vaccine or placebo; follow-up 72 weeks.	Extractable outcomes; used for evidence synthesis.	[9]
PXVX0317 / Vimkunya	CHIKV VLP vaccine	Registry/protocol	Phase III registry; healthy adults/adolescents; PXVX0317 versus placebo; Day 22 and Month 6 immunogenicity/safety objectives.	Protocol/registry; no extractable outcomes.	[10]
PXVX0317 / Vimkunya	CHIKV VLP vaccine	Registry/protocol	Phase II open-label registry; single adjuvanted PXVX0317 40 mcg; antibody assessed at D8, D15, and D22.	Protocol/registry; no extractable outcomes.	[11]
CHIKV VLP vaccine	CHIKV VLP vaccine	Conference abstract / early report	Phase II post hoc/long-term safety and immunogenicity context; parent VRC704 trial with two IM doses of 20 mcg CHIKV VLP 28 days apart.	Partial; limited details, retained for context.	[12]
PXVX0317 / Vimkunya	CHIKV VLP vaccine	Registry/protocol	Phase III registry; randomized, double-blind, placebo-controlled PXVX0317 study in adults aged >=65 years; follow-up 6 months.	Protocol/registry; no extractable outcomes.	[13]
PXVX0317 / Vimkunya	Alum-adjuvanted CHIKV VLP	Published clinical trial report	Phase II; n=415 randomized; age 18-45 years; USA; eight regimens, including adjuvanted, unadjuvanted, accelerated, single-dose, and booster schedules; follow-up approximately 2 years.	Extractable outcomes; used for evidence synthesis.	[14]
PXVX0317 / Vimkunya	CHIKV VLP vaccine	Registry/protocol	Long-term follow-up registry after single or booster PXVX0317 vaccination; no new primary vaccination schedule.	Protocol/registry; no extractable outcomes.	[15]
CHIKV VLP vaccine	CHIKV VLP vaccine	Conference abstract / early report	Phase II abstract; 60 adults: prior alphavirus vaccine recipients n=30 and naive controls n=30; single IM 40 mcg alum-adjuvanted CHIKV VLP.	Partial; preliminary or superseded by full publication.	[16]
VRC-CHKVLP059 / CHIKV VLP	CHIKV VLP vaccine	Secondary/pooled analysis	Phase II post hoc serostatus subgroup analysis; parent trial n=400; vaccine-recipient analysis by baseline serostatus; age 18-60 years; Caribbean endemic sites; two IM doses 28 days apart; follow-up 72 weeks.	Extractable outcomes; used for evidence synthesis.	[17]
PXVX0317 / Vimkunya	CHIKV VLP vaccine	Secondary/pooled analysis	Phase II secondary laboratory analysis; selected cohort n=20 vaccine recipients; parent PXVX0317 trial enrolled healthy adults age 18-45 years; samples through day 182.	Extractable outcomes; used for evidence synthesis.	[18]
CHIKV VLP vaccine	CHIKV VLP vaccine	Conference abstract / early report	Phase III conference abstract; two pivotal trials in participants aged >=12 years, including adults >=65 years; single IM CHIKV VLP vaccine or placebo; follow-up up to 4 years planned.	Partial; preliminary or superseded by full publications.	[19]
CHIKV VLP vaccine	CHIKV VLP vaccine	Conference abstract / early	Phase III abstract; n=3254 dosed: CHIKV VLP vaccine n=2790 and placebo n=464; age 12-64	Partial; preliminary or superseded by full publication.	[20]

Candidate/product	Platform	Record status	Study context	Handling / extractable data	Ref.
		report	years; single IM pre-filled syringe CHIKV VLP vaccine or placebo on Day 1; follow-up Day 183.		
CHIKV VLP vaccine	CHIKV VLP vaccine	Conference abstract / early report	Phase III abstract; n=413 randomized: vaccine n=206 and placebo n=207; age >=65 years; single IM CHIKV VLP vaccine or placebo on Day 1; follow-up Day 183.	Partial; preliminary or superseded by full publication.	[21]
CHIKV VLP vaccine	CHIKV VLP vaccine	Registry/protocol	Phase III pediatric registry; planned study in children 1 to <12 years; CHIKV VLP vaccine versus placebo; exact regimen not extractable from current record.	Protocol/registry; no extractable outcomes.	[22]
PXVX0317 / Vimkunya	Alum-adjuvanted CHIKV VLP	Published clinical trial report	Phase II; n=60; prior alphavirus vaccine recipients n=30 and matched vaccine-naive controls n=30; age 18-65 years; USA; single IM 40 mcg adjuvanted CHIKV VLP; follow-up Day 182.	Extractable outcomes; used for evidence synthesis.	[23]
PXVX0317 / Vimkunya	CHIKV VLP vaccine	Published clinical trial report	Phase III; n=3258 enrolled and n=3254 dosed; age 12-64 years; USA; single IM Vimkunya/PXVX0317 dose on study day 1; follow-up Day 183.	Extractable outcomes; used for evidence synthesis.	[24]
PXVX0317 / Vimkunya	CHIKV VLP vaccine	Published clinical trial report	Phase III; n=413 randomized; age >=65 years; USA; single IM Vimkunya/PXVX0317 dose or placebo on day 1; follow-up Day 183.	Extractable outcomes; used for evidence synthesis.	[25]
CHIKV VLP vaccine	CHIKV VLP vaccine	Registry/protocol	Phase 3b registry; randomized, double-blind, placebo-controlled efficacy, safety, and immunogenicity study of adjuvanted CHIKV VLP vaccine; regimen details NR.	Protocol/registry; no extractable outcomes.	[26]

MV-CHIK / V184 (n=14)

Candidate/product	Platform	Record status	Study context	Handling / extractable data	Ref.
MV-CHIK / V184	Measles-vector vaccine	Conference abstract / early report	Phase I/II abstract; n=42 healthy volunteers; three IM injections on days 0, 28, and 90; active/placebo-controlled dose-escalation study.	Partial; preliminary or superseded by full publication.	[27]
MV-CHIK / V184	Measles-vector vaccine	Published clinical trial report	Phase I; n=42 randomized; age 18-45 years; Austria; MV-CHIK low, medium, and high dose groups with booster on day 28 or day 90.	Extractable outcomes; used for evidence synthesis.	[28]
MV-CHIK / V184	Measles-vector vaccine	Registry/protocol	Phase I registry; up to 180 healthy adults planned; age 18-45 years; two IM doses; 5x10^4 or 5x10^5 TCID50; schedules Days 1/29, 1/85, or 1/169; follow-up 13 months.	Protocol/registry; no extractable outcomes.	[29]
MV-CHIK / V184	Measles-vector vaccine	Registry/protocol	Phase II registry; six treatment groups; age 18-55 years; low/high-dose MV-CHIK schedules, including day 0/28/196 and measles-prime subgroups; follow-up day 224.	Protocol/registry; no extractable outcomes.	[30]
MV-CHIK / V184	Measles-vector vaccine	Registry/protocol	Phase II registry; previously epidemic area; planned n=100; baseline seropositive and seronegative adults; randomized 4:1 to MV-CHIK versus MMR; two-dose schedule.	Protocol/registry; no extractable outcomes.	[31]
MV-CHIK / V184	Measles-vector vaccine	Conference abstract / early report	Phase II conference abstract; ongoing study in Austria/Germany; MV-CHIK versus placebo/control; exact regimen not extractable from abstract.	Partial; preliminary or superseded by full publication.	[32]
MV-CHIK / V184	Measles-vector vaccine	Registry/protocol	Registry record; healthy volunteers; MV-CHIK dose/formulation regimens with one or two vaccinations; long-term immunogenicity planned.	Protocol/registry; no extractable outcomes.	[33]
MV-CHIK / V184	Measles-vector vaccine	Conference abstract / early report	Phase II abstract; MV-CHIK induced functional neutralizing antibodies in 96% of subjects after two vaccinations.	Partial; preliminary or superseded by full publication.	[34]
MV-CHIK / V184	Measles-vector vaccine	Registry/protocol	Phase II registry; V184/MV-CHIK study in adults with prior CHIKV infection; two vaccinations 28 days apart versus saline placebo.	Protocol/registry; no extractable outcomes.	[35]
MV-CHIK / V184	Measles-vector vaccine	Conference abstract / early report	Phase II abstract; Puerto Rico; MV-CHIK in individuals with prior natural CHIKV infection; two IM doses; solicited AEs through 28 days after each dose and unsolicited AEs for 1 year.	Partial; limited details, retained for context.	[36]
MV-CHIK / V184	Measles-vector vaccine	Conference abstract / early report	Phase II abstract; n=263 healthy adults; Austria/Germany; dose/schedule comparison from same Phase II program as Reisinger 2019.	Partial; preliminary or superseded by full publication.	[37]
MV-CHIK / V184	Measles-vector vaccine	Published clinical trial report	Phase II; n=263 randomized; age 18-55 years; Austria/Germany; MV-CHIK 5x10^4 or 5x10^5 TCID50 with one or two immunizations; follow-up day 224.	Extractable outcomes; used for evidence synthesis.	[38]
MV-CHIK / V184	Measles-vector vaccine	Secondary/pooled analysis	Phase II secondary cellular-immunity analysis; subset n=12 MV-CHIK recipients; adults from parent Phase II trial; MV-CHIK 1x10^6 TCID50 IM on days 0 and 28; endpoint day 56.	Extractable outcomes; used for evidence synthesis.	[39]
MV-CHIK / V184	Measles-vector vaccine	Published clinical trial report	Phase I; n=180 enrolled; age 18-45 years; USA; two IM MV-CHIK doses (5x10^4 or 5x10^5 TCID50); second dose on day 29, 85, or 169.	Extractable outcomes; used for evidence synthesis.	[40]

VLA1553 / IXCHIQ (n=24)

Candidate/product	Platform	Record status	Study context	Handling / extractable data	Ref.
VLA1553 / IXCHIQ	Live attenuated vaccine	Registry/protocol	Phase I registry; 120 planned; age 18-45 years; single IM vaccination on Day 0; revaccination at Month 6 or Month 12; follow-up 13 months.	Protocol/registry; no extractable outcomes.	[41]
VLA1553 / IXCHIQ	Live attenuated vaccine	Conference abstract / early	Phase I abstract; n=120; age 18-45 years; single-shot VLA1553; three dose levels; Month 6	Partial; preliminary or superseded by full publication.	[42]

Candidate/product	Platform	Record status	Study context	Handling / extractable data	Ref.
		report	analysis planned.		
VLA1553 / IXCHIQ	Live attenuated vaccine	Registry/protocol	Phase III adolescent registry; n=754 randomized 2:1 VLA1553/placebo; age 12 to <18 years; single IM full dose VLA1553 1x10^4 TCID50 per dose on Day 1; follow-up Month 12.	Protocol/registry; no extractable outcomes.	[43]
VLA1553 / IXCHIQ	Live attenuated vaccine	Published clinical trial report	Phase I; n=120 randomized adults; age 18-45 years; USA; single IM dose on day 0 at low, medium, or high dose; revaccination at month 6 or month 12; follow-up 13 months.	Extractable outcomes; used for evidence synthesis.	[44]
VLA1553 / IXCHIQ	Live attenuated vaccine	Registry/protocol	Phase 3b registry; open-label persistence and long-term safety follow-up of adults rolled over from VLA1553-301; follow-up 2 years.	Protocol/registry; no extractable outcomes.	[45]
VLA1553 / IXCHIQ	Live attenuated vaccine	Registry/protocol	Phase III registry; healthy adults 18-45 years; single IM VLA1553 final dose; three lots randomized 1:1:1.	Protocol/registry; no extractable outcomes.	[46]
VLA1553 / IXCHIQ	Live attenuated vaccine	Conference abstract / early report	Phase III abstract; pivotal trial planned about 4,060 participants; randomized 3:1; adults; USA; single IM VLA1553 or placebo; follow-up 4 years.	Partial; preliminary or superseded by full publication.	[47]
VLA1553 / IXCHIQ	Live attenuated vaccine	Conference abstract / early report	Phase III abstract; ongoing pivotal phase 3; randomized 3:1; adult age strata; USA; single IM VLA1553 or placebo; early safety follow-up.	Partial; preliminary or superseded by full publication.	[48]
VLA1553 / IXCHIQ	Live attenuated vaccine	Registry/protocol	Phase II pediatric dose–response registry; children age 1-11 years; single VLA1553 dose-response design; follow-up 2 years.	Protocol/registry; no extractable outcomes.	[49]
VLA1553 / IXCHIQ	Live attenuated vaccine	Registry/protocol	Phase III registry; HIV-positive adults; open-label single final dose VLA1553.	Protocol/registry; no extractable outcomes.	[50]
VLA1553 / IXCHIQ	Live attenuated vaccine	Conference abstract / early report	Phase III abstract; adults; USA; follow-up 6 months / Day 180-183.	Partial; preliminary or superseded by full publication.	[51]
VLA1553 / IXCHIQ	Live attenuated vaccine	Published clinical trial report	Phase III; n=4128 randomized: VLA1553 n=3093, placebo n=1035; PP immunogenicity n=362; age >=18 years, including >=65 subgroup; USA; single IM VLA1553 or placebo; follow-up Day 180.	Extractable outcomes; used for evidence synthesis.	[52]
VLA1553 / IXCHIQ	Live attenuated vaccine	Secondary/pooled analysis	Pooled Phase III immunogenicity analysis; per-protocol VLA1553 n=656; adults >=18 years overall; pooled from VLA1553-301 and VLA1553-302; single IM dose; follow-up Day 180.	Extractable outcomes; used for evidence synthesis.	[53]
VLA1553 / IXCHIQ	Live attenuated vaccine	Safety-only / pooled safety	Pooled safety analysis; VLA1553 n=3520 and placebo n=1033; adults >=18 years; Phase I, pivotal Phase III, and lot-consistency trials; follow-up Day 180.	Safety-only; use for safety synthesis, not immunogenicity/durability.	[54]
VLA1553 / IXCHIQ	Live attenuated vaccine	Published clinical trial report	Phase III lot-to-lot consistency trial; n=409 randomized and n=408 vaccinated; PP immunogenicity n=362; age 18-45 years; USA; single IM dose; three manufacturing lots compared; follow-up Month 6.	Extractable outcomes; used for evidence synthesis.	[55]
VLA1553 / IXCHIQ	Live attenuated vaccine	Published clinical trial report	Phase 3b persistence study; n=363 analyzed; age >=18 years, including >=65 subgroup; USA; no new vaccination; antibody persistence after prior single VLA1553 dose; follow-up 2 years.	Extractable outcomes; used for evidence synthesis.	[56]
VLA1553 / IXCHIQ	Live attenuated vaccine	Registry/protocol	Phase III registry; at least n=3000 planned; children age 1-11 years; Latin America and/or Southeast Asia; single VLA1553 versus Nimenrix; planned follow-up 13 months.	Protocol/registry; no extractable outcomes.	[57]
VLA1553 + dengue vaccine	Live attenuated CHIKV vaccine co-administration study	Registry/protocol	Phase 3b co-administration registry; adults age 18-59 years; co-administration of live attenuated dengue vaccine and VLA1553 versus separate administration.	Protocol/registry; no extractable outcomes.	[58]
VLA1553 / IXCHIQ	Live attenuated vaccine	Published clinical trial report	Phase III adolescent interim study; n=754 vaccinated; age 12 to <18 years; Brazil; single IM VLA1553 1x10^4 TCID50/0.5 mL or placebo; interim follow-up Day 29.	Extractable outcomes; used for evidence synthesis.	[59]
VLA1553 / IXCHIQ	Live attenuated vaccine	Conference abstract / early report	Phase II pediatric dose–response abstract; children age 1-11 years; endemic countries; single-dose VLA1553 dose-response design; follow-up 12 months / 1 year.	Partial; preliminary or superseded by full publication.	[60]
VLA1553 / IXCHIQ	Live attenuated vaccine	Published clinical trial report	Phase III adolescent final study; n=765 randomized and n=754 vaccinated; age 12-17 years; Brazil; single IM VLA1553 1x10^4 TCID50/0.5 mL or placebo; follow-up 12 months.	Extractable outcomes; used for evidence synthesis.	[61]
VLA1553 / IXCHIQ	Live attenuated vaccine	Published clinical trial report	Phase 3b long-term follow-up; subset n=363 from pivotal Phase III trial; adults >=18 years, including >=65 subgroup; no new vaccination; follow-up 4 years.	Extractable outcomes; used for evidence synthesis.	[62]
VLA1553 / IXCHIQ	Live attenuated vaccine	Post-marketing safety	Post-authorization safety evaluation; approximately 55,900 IXCHIQ doses worldwide by 31 Aug 2025; 35 serious adverse events; real-world population; licensed use only.	Safety-only; use for safety synthesis, not immunogenicity/durability.	[63]
VLA1553 / IXCHIQ	Live attenuated vaccine	Published clinical trial report	Phase II pediatric study; n=304 children age 1-11 years; Dominican Republic and Honduras; single half-dose or full-dose VLA1553 versus Nimenrix; interim follow-up Day 29.	Extractable outcomes; used for evidence synthesis.	[64]

ChAdOx1 Chik (n=4)

Candidate/product	Platform	Record status	Study context	Handling / extractable data	Ref.
ChAdOx1 Chik	Simian adenoviral vector	Registry/protocol	Phase I registry; 24 volunteers; UK; single IM ChAdOx1 Chik dose at 5x10^9, 2.5x10^10, or	Protocol/registry; no extractable outcomes.	[65]

Candidate/product	Platform	Record status	Study context	Handling / extractable data	Ref.
			5x10^10 viral particles; follow-up 26 weeks / 6 months.		
ChAdOx1 Chik	Simian adenoviral vector	Conference abstract / early report	Phase I abstract; 24 participants received a single IM injection at three escalating doses; UK; follow-up 6 months.	Partial; preliminary or superseded by full publication.	[66]
ChAdOx1 Chik	Simian adenoviral vector	Registry/protocol	Phase Ib registry; ChAdOx1 Chik/Zika co-administration study; adults age 18-50 years; stepwise dose escalation; follow-up about 6 months.	Protocol/registry; no extractable outcomes.	[67]
ChAdOx1 Chik	Simian adenoviral vector	Published clinical trial report	Phase I; 24 healthy adults; three dose-escalation groups; age 18-50 years; United Kingdom; single IM ChAdOx1 Chik dose at 5x10^9, 2.5x10^10, or 5x10^10 viral particles; follow-up Day 182.	Extractable outcomes; used for evidence synthesis.	[68]

mRNA-1388 / VAL-181388 (n=3)

Candidate/product	Platform	Record status	Study context	Handling / extractable data	Ref.
mRNA-1388 / VAL-181388	mRNA vaccine	Registry/protocol	Phase I registry; randomized placebo-controlled dose-ranging study of VAL-181388 in healthy adults in a non-endemic region.	Protocol/registry; no extractable outcomes.	[69]
mRNA-1388 / VAL-181388	mRNA vaccine	Conference abstract / early report	Phase I abstract; n=60 total; 25, 50, or 100 mcg dose cohorts plus placebo; age 18-49 years; USA; IM injections at weeks 0 and 4; follow-up 1 year after last injection.	Partial; preliminary or superseded by full publication.	[70]
mRNA-1388 / VAL-181388	mRNA vaccine	Published clinical trial report	Phase I; n=60 randomized; age 18-49 years; USA non-endemic region; two IM injections 28 days apart; dose groups 25, 50, or 100 mcg, or placebo; follow-up 1 year after dose 2.	Extractable outcomes; used for evidence synthesis.	[71]

BBV87 (n=3)

Candidate/product	Platform	Record status	Study context	Handling / extractable data	Ref.
BBV87	Inactivated whole-virus vaccine	Registry/protocol	Phase I registry; 60 total planned; three test-dose groups, n=15, each plus placebo n=15; age 18-50 years; IM on Day 1, Day 29, and Day 57.	Protocol/registry; no extractable outcomes.	[72]
BBV87	Inactivated whole-virus vaccine	Registry/protocol	Phase II/III registry; planned n=3,210; age 12-65 years; Panama, Colombia, Guatemala, Costa Rica, and Thailand; BBV87 20 or 40 mcg; schedules 0-6 months or 0-28 days.	Protocol/registry; no extractable outcomes.	[73]
BBV87	Inactivated CHIKV vaccine	Registry/protocol	Phase II/III registry; planned n=3,210; age 12-65 years; BBV87 two-dose adaptive program; no posted results.	Protocol/registry; no extractable outcomes.	[74]

HydroVax-CHIK (n=1)

Candidate/product	Platform	Record status	Study context	Handling / extractable data	Ref.
HydroVax-CHIK	Inactivated CHIKV vaccine	Registry/protocol	Phase I registry; double-blind, randomized, placebo-controlled dose-escalation trial of inactivated CHIKV vaccine; exact dose groups NR in current record.	Protocol/registry; no extractable outcomes.	[75]

PepGNP-ChikV (n=1)

Candidate/product	Platform	Record status	Study context	Handling / extractable data	Ref.
PepGNP-ChikV	Synthetic nanoparticle/T-cell vaccine	Registry/protocol	Phase I registry; 40 planned; age 18-60 years; two injections 42 days apart; four dose cohorts; follow-up Day 407 / 365 days after last vaccination.	Protocol/registry; no extractable outcomes.	[76]

Other / out-of-scope record retained in the 77-reference library (n=1)

Candidate/product	Platform	Record status	Study context	Handling / extractable data	Ref.
mRNA anti-CHIKV mAb	mRNA-encoded monoclonal antibody	Non-active-vaccine intervention	Phase I trial of lipid-encapsulated mRNA encoding a monoclonal antibody with neutralizing activity against CHIKV; healthy adults age 18-50 years.	Out of scope for active vaccine candidate evidence; retained for audit only.	[77]

Supplementary Table S1B. Extractable immunogenicity and durability information, grouped by candidate/product.

Only records with extractable or partially extractable outcome information are shown below. Registry/protocol-only, safety-only, and out-of-scope records are documented in Table S1A.

TSI-GSD-218 (n=1)

Candidate/product	Record status	Study context (brief)	Extractable immunogenicity/durability information	Ref.
TSI-GSD-218	Published clinical trial report	Phase II; n=73 adults; age 18-40 years; USA; single 0.5 mL SC dose (~10^5 pfu) or placebo; follow-up 1 year.	Peak: Day 28 PRNT50 GMT 582; 57/58 evaluable vaccinees (98.3%) seroconverted by Day 28. M6: Day 180 sample collected, exact GMT NR. M12: Day 360 PRNT50 GMT 105; 85% remained seropositive.	[1]

CHIKV VLP program (VRC-CHKVLP059; PXVX0317/Vimkunya) (n=15)

Candidate/product	Record status	Study context (brief)	Extractable immunogenicity/durability information	Ref.
VRC-CHKVLP059 / VRC 311	Conference abstract / early report	Phase I; n=25; dose groups 10, 20, and 40 mcg; three IM doses on Days 0, 28, and 140.	Partial; preliminary or superseded by full publication.	[3]
VRC-CHKVLP059 / VRC 311	Published clinical trial report	Phase I; n=25; age 18-50 years; USA; three IM injections at weeks 0, 4, and 20; follow-up 44 weeks.	Peak: neutralization IC50 at week 24: 10 mcg 8745, 20 mcg 4525, 40 mcg 5390. M6-equivalent: week 44 IC50 940, 717, and 1385 for 10, 20, and 40 mcg groups.	[4]
CHIKV VLP vaccine	Conference abstract / early report	Cross-genotype neutralization analysis using sera from 12 VLP vaccine recipients.	Partial; broad neutralization across CHIKV genotypes reported; limited abstract-level detail.	[6]
VRC-CHKVLP059 / CHIKV VLP	Published clinical trial report	Phase II; n=400 randomized; age 18-60 years; Caribbean endemic sites; two IM doses 28 days apart; follow-up 72 weeks.	Peak: week 8 FRNT EC50 vaccine GMT 2004.5; CHIKV-luc NT80 peak in baseline-seronegative recipients week 8 GMT 1728. M6: week 24 NT80 211. M12: week 48 NT80 112. Longer follow-up: week 72 NT80 98.	[9]
CHIKV VLP vaccine	Conference abstract / early report	Phase II; parent VRC704 trial; two IM doses of 20 mcg CHIKV VLP 28 days apart; post hoc serostatus context.	Partial; limited details, retained for context.	[12]
PXVX0317 / Vimkunya	Published clinical trial report	Phase II; n=415 randomized; age 18-45 years; USA; eight regimens, including adjuvanted, unadjuvanted, accelerated, single-dose, and booster schedules; follow-up approximately 2 years.	Peak: Day 57 GMTs across groups 920.1-2057.0; single 40 mcg group GMT 1712.5; booster group GMT 10941.1 after the booster. M12: Day 365 GMTs 243.4, 336.6, and 491.7 in selected groups. Longer follow-up: Day 760 GMTs include 169.8 and 5365.1 after the booster.	[14]
CHIKV VLP vaccine	Conference abstract / early report	Phase II abstract; 60 adults; prior alphavirus vaccine recipients and naive controls; single IM 40 mcg alum-adjuvanted CHIKV VLP.	Partial; preliminary or superseded by full publication.	[16]
VRC-CHKVLP059 / CHIKV VLP	Secondary/pooled analysis	Phase II post hoc serostatus subgroup analysis; parent trial n=400; vaccine-recipient analysis by baseline serostatus; follow-up 72 weeks.	Peak: baseline-seropositive week 40 GMT 3594; baseline-seronegative week 8 GMT 1728. M6: week 24 GMT 2865 and 211. M12: week 48 GMT 2899 and 112. Longer follow-up: week 72 GMT 2993 and 98.	[17]
PXVX0317 / Vimkunya	Secondary/pooled analysis	Phase II secondary laboratory analysis; selected cohort n=20 vaccine recipients; samples through Day 182.	Peak: FRNT EC50 approximately 6800 at Day 29 and approximately 19000 at Day 57; similar LR2006 and RSU1 responses reported. M6: Day 182 EC50 approximately 1000 against CHIKV-37997.	[18]
CHIKV VLP vaccine	Conference abstract / early report	Phase III abstract; two pivotal trials in participants aged >=12 years, including adults >=65 years; follow-up planned.	Partial; preliminary or superseded by full publications.	[19]
CHIKV VLP vaccine	Conference abstract / early report	Phase III abstract; n=3254 dosed; age 12-64 years; single IM CHIKV VLP vaccine or placebo; follow-up Day 183.	Partial; preliminary or superseded by Richardson 2025 full publication.	[20]
CHIKV VLP vaccine	Conference abstract / early report	Phase III abstract; n=413 randomized; age >=65 years; single IM CHIKV VLP vaccine or placebo; follow-up Day 183.	Partial; preliminary or superseded by Tindale 2025 full publication.	[21]
PXVX0317 / Vimkunya	Published clinical trial report	Phase II; n=60; prior alphavirus vaccine recipients n=30 and matched vaccine-naive controls n=30; age 18-65 years; USA; single IM 40 mcg CHIKV VLP; follow-up Day 182.	Peak: Day 22 GMT 2032.5 in previous alphavirus vaccine recipients and 2299.2 in vaccine-naive controls. M6: Day 182 GMT 294.4 in previous alphavirus vaccine recipients; corresponding naive-control value reported in source.	[23]
PXVX0317 / Vimkunya	Published clinical trial report	Phase III; n=3258 enrolled; n=3254 dosed; age 12-64 years; USA; single IM Vimkunya/PXVX0317 dose on Study Day 1; follow-up Day 183.	Peak: baseline-seronegative recipients Day 22 GMT 1618; Day 15 GMT 1096. M6: Day 183 GMT 338 in baseline-seronegative recipients; seroresponse persisted to Month 6.	[24]
PXVX0317 / Vimkunya	Published clinical trial report	Phase III; n=413 randomized; age >=65 years; USA; single IM Vimkunya/PXVX0317 dose or placebo on Day 1; follow-up Day 183.	Peak: Day 22 GMT 724; seroresponse 165/189 (87%). M6: Day 183 GMT 233; seroresponse 139/184 (76%).	[25]

MV-CHIK / V184 (n=9)

Candidate/product	Record status	Study context (brief)	Extractable immunogenicity/durability information	Ref.
MV-CHIK / V184	Conference abstract / early report	Phase I/II abstract; n=42 healthy volunteers; three IM injections on days 0, 28, and 90.	Partial; preliminary or superseded by full publication.	[27]
MV-CHIK / V184	Published clinical trial report	Phase I; n=42 randomized; age 18-45 years; Austria; low, medium, and high dose groups with booster on day 28 or day	Day 28 PRNT50 seroconversion after one immunization: 44% low, 92% medium, 90% high; second vaccination led to 100% seroconversion in all candidate vaccine groups.	[28]

Candidate/product	Record status	Study context (brief)	Extractable immunogenicity/durability information	Ref.
		90.		
MV-CHIK / V184	Conference abstract / early report	Phase II abstract; Austria/Germany; MV-CHIK versus placebo/control; exact regimen NR.	Partial; preliminary or superseded by full publication.	[32]
MV-CHIK / V184	Conference abstract / early report	Phase II abstract; MV-CHIK induced functional neutralizing antibodies in 96% of subjects after two vaccinations.	Partial; preliminary or superseded by full publication.	[34]
MV-CHIK / V184	Conference abstract / early report	Phase II abstract; Puerto Rico; previously CHIKV-exposed and unexposed adults; two IM doses; safety-focused context.	Partial; limited details, retained for context.	[36]
MV-CHIK / V184	Conference abstract / early report	Phase II final-data abstract; n=263 healthy adults; Austria/Germany; dose/schedule comparison.	Partial; preliminary or superseded by Reisinger 2019 full publication.	[37]
MV-CHIK / V184	Published clinical trial report	Phase II; n=263 randomized; age 18-55 years; Austria/Germany; one or two immunizations with MV-CHIK 5x10^4 or 5x10^5 TCID50; follow-up day 224.	Peak: highest reported GMT 609.80 at Day 224 in high-dose group D after second immunization. Day 56 GMTs varied by group; Day 196 seroconversion reported.	[38]
MV-CHIK / V184	Secondary/pooled analysis	Phase II secondary cellular-immunity analysis; subset n=12 MV-CHIK recipients; adults from parent Phase II trial; endpoint Day 56.	Cellular-immunity endpoint: CHIKV-specific CD4+ T cells detected in 6/12 after one vaccination and 10/12 by Day 56 after two vaccinations.	[39]
MV-CHIK / V184	Published clinical trial report	Phase I; n=180 enrolled across six cohorts; age 18-45 years; USA; two IM MV-CHIK doses (5x10^4 or 5x10^5 TCID50); second dose on Day 29, 85, or 169.	Peak: high-dose Day 29 after second vaccination GMTs were 241.0 for D1/D29 interval and 678.1 for D1/D85 interval; higher titers with longer interval reported. Harmonized M6 GMT NR.	[40]

VLA1553 / IXCHIQ (n=14)

Candidate/product	Record status	Study context (brief)	Extractable immunogenicity/durability information	Ref.
VLA1553 / IXCHIQ	Conference abstract / early report	Phase I abstract; n=120; age 18-45 years; three dose levels; Month 6 analysis planned.	Partial; preliminary or superseded by full publication.	[42]
VLA1553 / IXCHIQ	Published clinical trial report	Phase I; n=120 randomized adults; age 18-45 years; USA; single IM dose at low, medium, or high dose; revaccination at Month 6 or Month 12.	Peak: Day 28 GMTs ranged from 592.6 to 686.9 across dose groups; 100% seroconversion by Day 14 and sustained to Month 12. M6: pre-revaccination GMT 452.5 in group H2. M12: GMTs after single vaccination ranged from 588.9 to 1005.8.	[44]
VLA1553 / IXCHIQ	Conference abstract / early report	Phase III abstract; pivotal trial planned; randomized 3:1; adults; USA; single IM VLA1553 or placebo; follow-up 4 years.	Partial; preliminary or superseded by full publication.	[47]
VLA1553 / IXCHIQ	Conference abstract / early report	Phase III abstract; early safety up to Day 29 from pivotal phase 3 program.	Partial; preliminary or superseded by full publication.	[48]
VLA1553 / IXCHIQ	Conference abstract / early report	Phase III abstract; adults; USA; follow-up 6 months / Day 180-183.	Partial; preliminary or superseded by full publication.	[51]
VLA1553 / IXCHIQ	Published clinical trial report	Phase III; n=4128 randomized; age >=18 years, including >=65 subgroup; USA; single IM VLA1553 or placebo; follow-up Day 180.	Peak: Day 29 GMT 3362; seroprotection 263/266 (98.9%) in baseline-negative VLA1553 recipients. M6: Day 180 GMT 752; 233/242 (96.3%) remained seroprotected.	[52]
VLA1553 / IXCHIQ	Secondary/pooled analysis	Pooled Phase III immunogenicity analysis; per-protocol VLA1553 n=656; adults >=18 years; single IM dose; follow-up Day 180.	Peak: Day 29 pooled GMT 2954.1; seroresponse 98.3%; seroconversion 98.5%. M6: Day 180 pooled GMT 735.3.	[53]
VLA1553 / IXCHIQ	Published clinical trial report	Phase III lot-to-lot consistency trial; n=408 vaccinated; PP immunogenicity n=362; age 18-45 years; USA; single IM dose; follow-up Month 6.	Peak: Day 28/29 GMT 2643; lot-to-lot consistency demonstrated. M6: Month 6 GMT 709.	[55]
VLA1553 / IXCHIQ	Published clinical trial report	Phase 3b persistence study; n=363 analyzed; adults >=18 years, including >=65 subgroup; no new vaccination; follow-up 2 years.	Persistence after prior single dose: Year 2 GMT about 785/819 depending on analysis set; seroresponse remained high through 2 years.	[56]
VLA1553 / IXCHIQ	Published clinical trial report	Phase III adolescent interim study; n=754 vaccinated; age 12 to <18 years; Brazil; single IM VLA1553 or placebo; interim follow-up Day 29.	Peak: Day 29 GMT approximately 3899/3856 in adolescents; high seroresponse after one dose; longer follow-up addressed in Buerger 2026.	[59]
VLA1553 / IXCHIQ	Conference abstract / early report	Phase II pediatric dose–response abstract; children age 1-11 years; single-dose VLA1553; follow-up 12 months planned.	Partial; preliminary or superseded by full publication.	[60]
VLA1553 / IXCHIQ	Published clinical trial report	Phase III adolescent final study; n=765 randomized and n=754 vaccinated; age 12-17 years; Brazil; single IM VLA1553 or placebo; follow-up 12 months.	Peak: Day 28 GMT 3856. M6: Month 6 GMT 1360. M12: Month 12 GMT 1284.	[61]

Candidate/product	Record status	Study context (brief)	Extractable immunogenicity/durability information	Ref.
VLA1553 / IXCHIQ	Published clinical trial report	Phase 3b long-term follow-up; subset n=363 from pivotal Phase III trial; adults >=18 years, including >=65 subgroup; no new vaccination; follow-up 4 years.	Peak: PP table total Day 29 GMT 3762.0. M6: Day 180 GMT 1019.1. M12: Year 1 GMT 1070.3. Longer follow-up: Year 2 GMT 819.5, Year 3 GMT 748.0, Year 4 GMT 609.8.	[62]
VLA1553 / IXCHIQ	Published clinical trial report	Phase II pediatric study; n=304 children age 1-11 years; Dominican Republic and Honduras; half-dose or full-dose VLA1553 versus Nimenrix; interim follow-up Day 29.	Peak: Day 29 full-dose GMTs by age group were 3443.6 (7-11 years), 3134.8 (3-6 years), and 2161.7 (1-2 years). M6/M12: not available in interim analysis.	[64]

ChAdOx1 Chik (n=2)

Candidate/product	Record status	Study context (brief)	Extractable immunogenicity/durability information	Ref.
ChAdOx1 Chik	Conference abstract / early report	Phase I abstract; 24 participants received a single IM injection at three escalating doses; UK; follow-up 6 months.	Partial; preliminary or superseded by full publication.	[66]
ChAdOx1 Chik	Published clinical trial report	Phase I; 24 healthy adults; three dose-escalation groups; age 18-50 years; UK; single IM ChAdOx1 Chik dose.	Peak PRNT50 GMTs: IOL Day 28 285.1; West African Day 28 369.7; Asian Day 56 75.3; Asian/American Day 28 71.3. M6: Day 182 PRNT50 213.6 (IOL), 229.7 (West African), and 95.1 (Asian).	[68]

mRNA-1388 / VAL-181388 (n=2)

Candidate/product	Record status	Study context (brief)	Extractable immunogenicity/durability information	Ref.
mRNA-1388 / VAL-181388	Conference abstract / early report	Phase I abstract; n=60; age 18-49 years; USA; IM injections at weeks 0 and 4; follow-up 1 year after last injection.	Partial; preliminary or superseded by full publication.	[70]
mRNA-1388 / VAL-181388	Published clinical trial report	Phase I; n=60 randomized; age 18-49 years; USA non-endemic region; two IM doses 28 days apart; dose groups 25, 50, or 100 mcg; follow-up 1 year after dose 2.	Peak: Day 56 GMTs after dose 2 were 6.2 (25 mcg), 53.8 (50 mcg), and 92.8 (100 mcg); placebo 5.0. M6 and M12 exact GMTs NR in main text/figure extraction; responses persisted in higher-dose groups.	[71]

1. Edelman, R.; Tacket, C.O.; Wasserman, S.S.; Bodison, S.A.; Perry, J.G.; Mangiafico, J.A. Phase II safety and immunogenicity study of live chikungunya virus vaccine TSI-GSD-218. *American Journal of Tropical Medicine and Hygiene* 2000, *62*, 681-685, doi:10.4269/ajtmh.2000.62.681.

2. VRC 311: A Phase 1 Open Label, Dose-Escalation Clinical Trial to Evaluate the Safety and Immunogenicity of a Virus-Like Particle (VLP) Chikungunya Vaccine, VRC-CHKVLP059-00-VP, in Healthy Adults. 2011.

3. Chang, L.J.; Mendoza, F.; Saunders, J.; Plummer, S.; Yamshchikov, G.V.; Ledgerwood, J.E.; Graham, B.S. VRC 311: Phase i clinical trial of a virus-like particle chikungunya vaccine in healthy adults. *Journal of Allergy and Clinical Immunology* 2013, *131*, AB330, doi:10.1016/j.jaci.2012.12.1555.

4. Chang, L.J.; Dowd, K.A.; Mendoza, F.H.; Saunders, J.G.; Sitar, S.; Plummer, S.H.; Yamshchikov, G.; Sarwar, U.N.; Hu, Z.; Enama, M.E.; et al. Safety and tolerability of chikungunya virus-like particle vaccine in healthy adults: A phase 1 dose-escalation trial. *The Lancet* 2014, *384*, 2046-2052, doi:10.1016/S0140-6736(14)61185-5.

5. Phase 2 Randomized, Placebo-Controlled Trial to Evaluate the Safety and Immunogenicity of a Chikungunya Virus-Like Particle Vaccine, VRC-CHKVLP059-00-VP, in Healthy Adults. 2015.

6. Goo, L.; Dowd, K.A.; Lin, T.Y.; Graham, B.S.; Ledgerwood, J.E.; Pierson, T.C. A virus-like particle vaccine elicits broad neutralizing antibody responses in humans against distinct chikungunya virus genotypes. *American Journal of Tropical Medicine and Hygiene* 2016, *95*, 199, doi:10.4269/ajtmh.abstract2016.

7. A Phase 2 Parallel-Group, Randomized, Double-Blind Study to Assess the Safety and Immunogenicity of PXVX0317 (Chikungunya Virus Virus-Like Particle Vaccine [CHIKV-VLP], Unadjuvanted or Alum-adjuvanted). 2018.

8. A Phase 2 Open-label Study to Assess the Safety and Immunogenicity of an Alum-adjuvanted Chikungunya Virus-like Particle Vaccine (PXVX0317) in Prior Recipients of Other Alphavirus Vaccines Versus Alphavirus Naïve Controls. 2019.

9. Chen, G.L.; Coates, E.E.; Plummer, S.H.; Carter, C.A.; Berkowitz, N.; Conan-Cibotti, M.; Cox, J.H.; Beck, A.; O'Callahan, M.; Andrews, C.; et al. Effect of a Chikungunya Virus-Like Particle Vaccine on Safety and Tolerability Outcomes A Randomized Clinical Trial. *Jama-Journal of the American Medical Association* 2020, *323*, 1369-1377, doi:10.1001/jama.2020.2477.

10. A Phase 3 Safety, Immunogenicity, and Lot-Consistency Trial of the VLP-Based Chikungunya Vaccine PXVX0317 in Healthy Adults and Adolescents. 2021.

11. A Phase 2 Open-Label Study to Assess the Safety and Immunogenicity of PXVX0317 (Chikungunya Virus Virus-Like Particle Vaccine [CHIKV VLP], Aluminum Hydroxide Adjuvanted). 2021.

12. Ramanathan, R.; Ledgerwood, J.; Coates, E.; Mendy, J.; Bedell, L.; Richardson, J.; Prithviraj, R.; Tredo, S.R.; Warfield, K.L.; Cabell, C. LONG-TERM SAFETY AND IMMUNOGENICITY OF CHIKUNGUNYA VIRUS-LIKE PARTICLE VACCINE IN CHIKUNGUNYA SEROPOSITIVE INDIVIDUALS. *American Journal of Tropical Medicine and Hygiene* 2021, *105*, 272, doi:10.4269/ajtmh.abstract2021.

13. A Phase 3 Safety and Immunogenicity Trial of the VLP-Based Chikungunya Virus Vaccine PXVX0317 in Adults ≥65 Years of Age. 2022.

14. Bennett, S.R.; McCarty, J.M.; Ramanathan, R.; Mendy, J.; Richardson, J.S.; Smith, J.; Alexander, J.; Ledgerwood, J.E.; de Lame, P.A.; Royalty Tredo, S.; et al. Safety and immunogenicity of PXVX0317, an aluminium hydroxide-adjuvanted chikungunya virus-like particle vaccine: a randomised, double-blind, parallel-group, phase 2 trial. *The Lancet Infectious Diseases* 2022, *22*, 1343-1355, doi:10.1016/S1473-3099(22)00226-2.

15. A Long-term Follow-up Study to Evaluate Safety and Immunogenicity of a Chikungunya Virus Virus-like Particle Vaccine (PXVX0317) in Healthy Adults and Adolescents After Either a Single or Booster Vaccination Dosing Regimen. 2023.

16. Hamer, M.J.; Pierson, B.; Haller, J.; Lee, C.; Hutter, J.N.; Martins, K.; Glass, P.; Liggett, D.; Sanborn, A.; Moon, J.E.; et al. A PHASE 2 OPEN-LABEL STUDY TO ASSESS THE SAFETY AND IMMUNOGENICITY OF AN ALUM-ADJUVANTED CHIKUNGUNYA VIRUS-LIKE PARTICLE (VLP) VACCINE IN PRIOR RECIPIENTS OF OTHER ALPHAVIRUS VACCINES VERSUS ALPHAVIRUS NAÏVE CONTROLS. *American Journal of Tropical Medicine and Hygiene* 2023, *108*, 214.

17. McCarty, J.M.; Bedell, L.; Mendy, J.; Coates, E.E.; Chen, G.L.; Ledgerwood, J.E.; Tredo, S.R.; Warfield, K.L.; Richardson, J.S. Chikungunya virus virus-like particle vaccine is well tolerated and immunogenic in chikungunya seropositive individuals. *Vaccine* 2023, *41*, 6146-6149, doi:10.1016/j.vaccine.2023.08.086.

18. Raju, S.; Adams, L.J.; Earnest, J.T.; Warfield, K.; Vang, L.; Crowe, J.E.; Fremont, D.H.; Diamond, M.S. A chikungunya virus-like particle vaccine induces broadly neutralizing and protective antibodies against alphaviruses in humans. *Science Translational Medicine* 2023, *15*, doi:10.1126/scitranslmed.ade8273.

19. Richardson, J.S.; Anderson, D.; Mendy, J.; Muhammad, S.; Tindale, L.; Loreth, T.; Tredo, S.R.; Jenkins, V.; Ajiboye, P.; Bedell, L. Safety and Immunogenicity of an Adjuvanted Chikungunya Virus (CHIKV) Virus-like Particle (VLP) Based Vaccine in Two Pivotal Phase 3 Trials, ≥12 Years of Age. *Open Forum Infectious Diseases* 2023, *10*, S1270-S1271, doi:10.1093/ofid/ofad500.2471.

20. Richardson, J.S.; Anderson, D.M.; Mendy, J.; Tindale, L.C.; Muhammad, S.; Loreth, T.; Tredo, S.R.; Warfield, K.L.; Ramanathan, R.; Caso, J.T.; et al. Chikungunya Virus VLP Vaccine: Phase 3 Trial in Adolescents and Adults. 2024, doi:10.1101/2024.10.11.24315179.

21. Tindale, L.C.; Richardson, J.S.; Anderson, D.M.; Mendy, J.; Muhammad, S.; Loreth, T.; Tredo, S.R.; Ramanathan, R.; Jenkins, V.A.; Bedell, L.; et al. Chikungunya Virus VLP Vaccine: Phase 3 Trial in Adults ≥65 Years of Age. 2024, doi:10.1101/2024.10.10.24315205.

22. A Phase 3 Global, Randomized, Double-Blind, Placebo-Controlled, Safety and Immunogenicity Study of CHIKV VLP Vaccine in Children 1 to <12 Years of Age. 2025.

23. Hamer, M.J.; McCarty, J.M.; Pierson, B.C.; Regules, J.A.; Mendy, J.; Sanborn, A.; Gardner, C.L.; Haller, J.M.; Gregory, M.K.; Liggett, D.L.; et al. Safety and immunogenicity of an adjuvanted chikungunya virus virus-like particle (CHIKV VLP) vaccine in previous recipients of other alphavirus vaccines versus alphavirus vaccine-naive controls: an open-label, parallel-group, age-matched, sex-matched, phase 2 randomised controlled study. *Lancet Microbe* 2025, *6*, doi:10.1016/j.lanmic.2024.101000.

24. Richardson, J.S.; Anderson, D.M.; Mendy, J.; Tindale, L.C.; Muhammad, S.; Loreth, T.; Tredo, S.R.; Warfield, K.L.; Ramanathan, R.; Caso, J.; et al. Chikungunya virus virus-like particle vaccine safety and immunogenicity in adolescents and adults in the USA : a phase 3, randomised, double-blind, placebo-controlled trial. *Lancet* 2025, *405*, 1343-1352, doi:10.1016/s0140-6736(25)00345-9.

25. Tindale, L.C.; Richardson, J.S.; Anderson, D.M.; Mendy, J.; Muhammad, S.; Loreth, T.; Tredo, S.R.; Ramanathan, R.; Jenkins, V.A.; Bedell, L.; et al. Chikungunya virus virus-like particle vaccine safety and immunogenicity in adults older than 65 years: a phase randomised, double-blind, placebo-controlled trial. *Lancet* 2025, *405*, 1353-1361, doi:10.1016/s0140-6736(25)00372-1.

26. A Phase 3b Randomized, Double-blind, Placebo-controlled Study to Evaluate the Efficacy, Safety, and Immunogenicity of an Adjuvanted Chikungunya Virus Virus-like Particle (CHIKV VLP) Vaccine for the Prevention of Chikungunya Disease in Adolescents (12 to <18 Years) and Adults (≥18 Years). 2026.

27. Ramsauer, K.; Despres, P.; Firbas, C.; Müllner, M.; Tangy, F.; Tauber, E. Clinical results of novel chikungunya vaccine tested in phase 1/2 trial: Neutralizing antibodies and anti-vector immunity. *American Journal of Tropical Medicine and Hygiene* 2014, *91*, 4.

28. Ramsauer, K.; Schwameis, M.; Firbas, C.; Müllner, M.; Putnak, R.J.; Thomas, S.J.; Desprès, P.; Tauber, E.; Jilma, B.; Tangy, F. Immunogenicity, safety, and tolerability of a recombinant measles-virus-based chikungunya vaccine: A randomised, double-blind, placebo-controlled, active-comparator, first-in-man trial. *The Lancet Infectious Diseases* 2015, *15*, 519-527, doi:10.1016/S1473-3099(15)70043-5.

29. A Phase 1, Double Blinded, Placebo Controlled, Dose Comparison Trial to Evaluate the Safety, Immunogenicity and Schedule of Measles-Vectored Chikungunya Virus Vaccine (MV-CHIK) in Healthy Adults. 2016.

30. Double Blinded, Randomized, Priorix®- and Placebo-controlled, Trial to Evaluate the Optimal Dose of MV-CHIK Vaccine (Against Chikungunya Virus) in Regard to Immunogenicity, Safety and Tolerability in Healthy Volunteers. 2016.

31. Phase 2 Study of a Live Attenuated Measles Virus-Vectored Chikungunya Vaccine in a Previously Epidemic Area. 2017.

32. Schrauf, S.; Ramsauer, K.; Müllner, M.; Pfeiffer, A.; Kort, A.; Tauber, E. Safety and immunogenicity of a live recombinant measles vector based chikungunya vaccine in healthy adults: A randomized, placebo controlled phase 2 study. *American Journal of Tropical Medicine and Hygiene* 2017, *97*, 1.

33. Observer Blinded, Randomised Study to Investigate Safety, Tolerability and Long-term Immunogenicity of Different Dose Regimens and Formulations of MV-CHIK in Healthy Volunteers. 2018.

34. Ramsauer, K.; Reisinger, E.; Firbas, C.; Wiedermann-Schmidt, U.; Beubler, E.; Aberle, J.; Müllner, M.; Pfeiffer, A.; Vielnascher, R.; Tauber, E. Phase 2 clinical results - Chikungunya vaccine based on measles vector (MV-CHIK) induces humoral and cellular responses in the presence of pre-existing anti measles immunity. *American Journal of Tropical Medicine and Hygiene* 2018, *99*, 202-203.

35. Phase 2 Study of a Live Attenuated Measles Virus-Vectored Chikungunya Vaccine in Previously Exposed Adults. 2019.

36. Ramsauer, K.; Diaz, C.; Febo, I.; Powell, J.; Maldonado, A.R.; Vielnascher, R.; Keiser, P.B. Safety of the measles-vectored chikungunya vaccine (MV-chik) in healthy volunteers previously exposed to chikungunya virus. *American Journal of Tropical Medicine and Hygiene* 2019, *101*, 18, doi:10.4269/ajtmh.abstract2019.

37. Ramsauer, K.; Reisinger, E.; Firbas, C.; Wiedermann-Schmidt, U.; Beubler, E.; Pfeiffer, A.; Müllner, M.; Aberle, J.; Tauber, E. Phase 2 clinical results: Chikungunya vaccine based on measles vector (MV-CHIK) induces humoral and cellular responses in the presence of pre-existing anti measles immunity. *International Journal of Infectious Diseases* 2019, *79*, 118, doi:10.1016/j.ijid.2018.11.291.

38. Reisinger, E.C.; Tschismarov, R.; Beubler, E.; Wiedermann, U.; Firbas, C.; Loebermann, M.; Pfeiffer, A.; Muellner, M.; Tauber, E.; Ramsauer, K. Immunogenicity, safety, and tolerability of the measles-vectored chikungunya virus vaccine MV-CHIK: a double-blind, randomised, placebo-controlled and active-controlled phase 2 trial. *Lancet* 2019, *392*, 2718-2727, doi:10.1016/s0140-6736(18)32488-7.

39. Schmitz, K.S.; Comvalius, A.D.; Nieuwkoop, N.J.; Geers, D.; Weiskopf, D.; Ramsauer, K.; Sette, A.; Tschismarov, R.; de Vries, R.D.; de Swart, R.L. A measles virus-based vaccine induces robust chikungunya virus-specific CD4+ T-cell responses in a phase II clinical trial. *Vaccine* 2023, *41*, 6495-6504, doi:10.1016/j.vaccine.2023.09.022.

40. Winokur, P.; Hegmann, T.E.; El Sahly, H.M.; Anderson, E.J.; Grp, D.S. A Phase 1 Double-Blinded Trial to Evaluate Safety, Immunogenicity, and Dosing of Measles-Vectored Chikungunya Virus Vaccine (MV-CHIK) in Healthy Adults. *Journal of Infectious Diseases* 2026, *233*, e641-e645, doi:10.1093/infdis/jiaf571.

41. A Randomized, Observer-Blinded, Dose-Escalation Phase 1 Study to Assess the Safety and Immunogenicity of Three Different Dose Levels of a Live-Attenuated Chikungunya Virus Vaccine Candidate (VLA1553) in Healthy Volunteers Aged 18 To 45 Years. 2017.

42. Wressnigg, N.; Hochreiter, R.; Fritzer, A.; Schlegel, R.; Meinke, A. Chikungunya: Phase 1 clinical development of a single-shot live-attenuated vaccine. *American Journal of Tropical Medicine and Hygiene* 2019, *101*, 19-20, doi:10.4269/ajtmh.abstract2019.

43. A Multicenter, Randomized, Controlled, Double Blinded Pivotal Study to Evaluate Safety and Immunogenicity of a Live-attenuated Chikungunya Virus Vaccine Candidate (VLA1553) in Adolescents Aged 12 Years to <18 Years. 2020.

44. Wressnigg, N.; Hochreiter, R.; Zoihsl, O.; Fritzer, A.; Bézay, N.; Klingler, A.; Lingnau, K.; Schneider, M.; Lundberg, U.; Meinke, A.; et al. Single-shot live-attenuated chikungunya vaccine in healthy adults: a phase 1, randomised controlled trial. *The Lancet Infectious Diseases* 2020, *20*, 1193-1203, doi:10.1016/S1473-3099(20)30238-3.

45. An Open-Label, Single Arm Trial To Evaluate Antibody Persistence And Long Term Safety Of A Live-Attenuated Chikungunya Virus Vaccine (VLA1553) In Adults Aged 18 Years and Above. 2021.

46. A Randomized, Double-Blinded Phase 3 Study to Demonstrate Lot-to-Lot Consistency of Three Lots of a Live-Attenuated Chikungunya Virus Vaccine Candidate (VLA1553) in Healthy Adults Aged 18 to 45 Years. 2021.

47. Jaramillo, J.C.; Schneider, M.; Narciso, M.; Hochreiter, R.; Bitzer, A.; Mader, R.; Dubischar, K.; Zoihsl, O.; Wressnigg, N.; Eder-Lingelbach, S. CHIKUNGUNYA: PHASE 3 CLINICAL DEVELOPMENT OF A SINGLE-SHOT LIVE-ATTENUATED VACCINE. *American Journal of Tropical Medicine and Hygiene* 2021, *105*, 271-272, doi:10.4269/ajtmh.abstract2021.

48. Schneider, M.; Narciso, M.; Hadl, S.; McMahon, R.; Toepfer, S.; Mader, R.; Jaramillo, J.C.; Dubischar, K.; Zoihsl, O.; Wressnigg, N.; et al. CHIKUNGUNYA: SAFETY UP TO DAY 29 OF PHASE 3 CLINICAL DEVELOPMENT OF A SINGLE-SHOT LIVE-ATTENUATED VACCINE. *American Journal of Tropical Medicine and Hygiene* 2021, *105*, 45-46, doi:10.4269/ajtmh.abstract2021.

49. A Randomized, Observer-blinded, Dose Response Phase 2 Trial to Assess the Safety and Immunogenicity of Two Different Dose Levels of a Live-attenuated Chikungunya Virus Vaccine (VLA1553) in Healthy Children Aged 1 to 11 Years. 2023.

50. An Open-label Phase 3 Trial to Assess the Safety and Immunogenicity of a Live-attenuated Chikungunya Virus Vaccine (VLA1553) in Moderately Immunocompromised Adult Participants Infected with Human Immunodeficiency Virus. 2023.

51. Bürger, V.; Schneider, M.; Hadl, S.; Narciso, M.; McMahon, R.; Töpfer, S.; Fuchs, U.; Hochreiter, R.; Bitzer, A.; Kosulin, K.; et al. CHIKUNGUNYA: PHASE 3 CLINICAL DEVELOPMENT OF A SINGLE-SHOT LIVE-ATTENUATED VACCINE. *American Journal of Tropical Medicine and Hygiene* 2023, *108*, 214.

52. Schneider, M.; Narciso-Abraham, M.; Hadl, S.; McMahon, R.; Toepfer, S.; Fuchs, U.; Hochreiter, R.; Bitzer, A.; Kosulin, K.; Larcher-Senn, J.; et al. Safety and immunogenicity of a single-shot live-attenuated chikungunya vaccine: a double-blind, multicentre, randomised, placebo-controlled, phase 3 trial. *The Lancet* 2023, *401*, 2138-2147, doi:10.1016/S0140-6736(23)00641-4.

53. Buerger, V.; Maurer, G.; Kosulin, K.; Hochreiter, R.; Larcher-Senn, J.; Dubischar, K.; Eder-Lingelbach, S. Combined immunogenicity evaluation for a new single-dose live-attenuated chikungunya vaccine. *Journal of Travel Medicine* 2024, *31*, doi:10.1093/jtm/taae084.

54. Maurer, G.; Buerger, V.; Larcher-Senn, J.; Erlsbacher, F.; Dubischar, K.; Eder-Lingelbach, S.; Jaramillo, J.C. Pooled safety evaluation for a new single-shot live-attenuated chikungunya vaccine. *Journal of Travel Medicine* 2024, *31*, doi:10.1093/jtm/taae133.

55. McMahon, R.; Fuchs, U.; Schneider, M.; Hadl, S.; Hochreiter, R.; Bitzer, A.; Kosulin, K.; Koren, M.; Mader, R.; Zoihsl, O.; et al. A randomized, double-blinded Phase 3 study to demonstrate lot-to-lot consistency and to confirm immunogenicity and safety of the live-attenuated chikungunya virus vaccine candidate VLA1553 in healthy adults. *Journal of Travel Medicine* 2024, *31*, doi:10.1093/jtm/taad156.

56. McMahon, R.; Toepfer, S.; Sattler, N.; Schneider, M.; Narciso-Abraham, M.; Hadl, S.; Hochreiter, R.; Kosulin, K.; Mader, R.; Zoihsl, O.; et al. Antibody persistence and safety of a live-attenuated chikungunya virus vaccine up to 2 years after single-dose administration in adults in the USA: a single-arm multicentre, phase 3b study. *Lancet Infectious Diseases* 2024, *24*, 1383-1392, doi:10.1016/s1473-3099(24)00357-8.

57. A Multicenter, Randomized, Controlled, Double-blind Pivotal Phase 3 Trial to Evaluate the Safety and Immunogenicity of a Live-attenuated Chikungunya Virus Vaccine (VLA1553) in Healthy Children Aged 1 to 11 Years. 2025.

58. Phase 3b, Multicenter, Randomized, Controlled, Double-Blind Clinical Trial to Evaluate the Immunogenicity and Safety of the Co-administration of Live Attenuated Dengue and Chikungunya Vaccines in Adults Aged 18 to 59 Years. 2025.

59. Buerger, V.; Hadl, S.; Schneider, M.; Schaden, M.; Hochreiter, R.; Bitzer, A.; Kosulin, K.; Mader, R.; Zoihsl, O.; Pfeiffer, A.; et al. Safety and immunogenicity of a live-attenuated chikungunya virus vaccine in endemic areas of Brazil: interim results of a double-blind, randomised, placebo-controlled phase 3 trial in adolescents. *The Lancet Infectious Diseases* 2025, *25*, 114-125, doi:10.1016/S1473-3099(24)00458-4.

60. Bürger, V.; Weisova, P.; Benedek, P.; Schneider, M.; Fuchs, U.; Hochreiter, R.; Bitzer, A.; Kosulin, K.; Zoihsl, O.; Dubischar, K.; et al. CHIKUNGUNYA: ONGOING DOSE-RESPONSE, SAFETY, AND IMMUNOGENICITY PHASE 2 TRIAL OF SINGLE-DOSE LIVE-ATTENUATED VACCINE (VLA1553) IN CHILDREN AGED 1 TO 11 YEARS. *American Journal of Tropical Medicine and Hygiene* 2025, *112*, 593.

61. Buerger, V.; Pfeiffer, A.; Schoengrundner, P.; Seebacher, J.; Hochreiter, R.; Kosulin, K.; Zoihsl, O.; Weisova, P.; Mader, R.; Loch, A.P.; et al. Safety and immunogenicity of a live-attenuated chikungunya virus vaccine in adolescents: final results from a 12-month, double-blind, randomised, placebo-controlled, phase 3 trial in endemic areas of Brazil. *Lancet Infect Dis* 2026, *26*, 417-428, doi:10.1016/s1473-3099(25)00631-0.

62. Sattler, N.; Scheiblauer, S.; Hochreiter, R.; Kosulin, K.; Buerger, V. Chikungunya virus-neutralizing antibody persistence four years after single-dose vaccination with VLA1553 (IXCHIQ®). *Vaccine* 2026, *88*, doi:10.1016/j.vaccine.2026.128787.

63. Vondeling, G.T.; Croda, J.; Jelinek, T.; Kassianos, G.; Kollaritsch, H.; Nguyen, L.B.L.; Ribeiro, G.S.; Schmidt-Chanasit, J.; Thomas, S.J.; Unger, Z.; et al. Post-marketing safety evaluation of the live-attenuated chikungunya vaccine (IXCHIQ). *Vaccine* 2026, *79*, doi:10.1016/j.vaccine.2026.128491.

64. Weisová, P.; Scheiblauer, S.; Ecker, J.; Schneider, M.; Hochreiter, R.; Bitzer, A.; Kosulin, K.; Schoengrundner, P.; Fuchs, U.; Rodeles, L.; et al. Live-attenuated chikungunya vaccine in children: a randomized phase 2 trial. *Nature Medicine* 2026, *32*, 561-571, doi:10.1038/s41591-025-04197-2.

65. A Phase I Study to Determine the Safety and Immunogenicity of the Candidate Chikungunya Virus (CHIKV) Vaccine ChAdOx1 Chik in Healthy Adult Volunteers. 2018.

66. Folegatti, P.M.; Harrison, K.; Lopez, F.R.; Tilley, M.W.; Lopez-Camacho, C.; Kim, Y.C.; Preciado-Llanes, L.; Rossi, S.L.; Poulton, I.; Jenkin, D.; et al. Safety and immunogenicity of a replication deficient simian adenoviral vectored chikungunya vaccine: A phase i, first-in-human, dose escalation trial. *American Journal of Tropical Medicine and Hygiene* 2019, *101*, 19, doi:10.4269/ajtmh.abstract2019.

67. A Single Centre, Double-blind, Double-dummy Placebo-controlled, Randomised Phase Ib Study to Evaluate the Safety & Immunogenicity of the Candidate Chikungunya Vaccine ChAdOx1 Chik & the Zika Vaccine ChAdOx1 Zika in Healthy Adults in Mexico. 2020.

68. Folegatti, P.M.; Harrison, K.; Preciado-Llanes, L.; Lopez, F.R.; Bittaye, M.; Kim, Y.C.; Flaxman, A.; Bellamy, D.; Makinson, R.; Sheridan, J.; et al. A single dose of ChAdOx1 Chik vaccine induces neutralizing antibodies against four chikungunya virus lineages in a phase 1 clinical trial. *Nature Communications* 2021, *12*, doi:10.1038/s41467-021-24906-y.

69. A Phase 1, Randomized, Placebo-Controlled, Dose-Ranging Study to Evaluate the Safety and Immunogenicity of VAL-181388 in Healthy Adults in a Non-endemic Chikungunya Region. 2017.

70. Shaw, C.; Panther, L.; August, A.; Zaks, T.; Smolenov, I.; Bart, S.; Watson, M. Safety and immunogenicity of a mRNA-based chikungunya vaccine in a phase 1 dose-ranging trial. *International Journal of Infectious Diseases* 2019, *79*, 17, doi:10.1016/j.ijid.2018.11.058.

71. Shaw, C.A.; August, A.; Bart, S.; Booth, P.G.J.; Knightly, C.; Brasel, T.; Weaver, S.C.; Zhou, H.; Panther, L. A phase 1, randomized, placebo-controlled, dose-ranging study to evaluate the safety and immunogenicity of an mRNA-based chikungunya virus vaccine in healthy adults. *Vaccine* 2023, *41*, 3898-3906, doi:10.1016/j.vaccine.2023.04.064.

72. Phase-I Open Label, Dose-escalation Clinical Trial to Evaluate the Safety, Tolerability and Immunogenicity of Chikungunya Vaccine in Healthy Adults of 18 to 50 Years Age. 2017.

73. A Phase II/III Adaptive Seamless Design, Randomized, Controlled Trial To Evaluate Safety And Immunogenicity of 2 Dose-Regimen of BBV87 Chikungunya Vaccine In Healthy Subjects Aged 12 to 65 Years in Latin America and Asia. 2020.

74. A Seamless Phase II/III, Observer-blind, Multi-centre, Randomized Clinical Trial to Evaluate Immunogenicity and Safety of BBV87, an Inactivated Chikungunya Virus Vaccine in Healthy Subjects 12-65 Years of Age. 2026.

75. A Double Blind, Randomized, Placebo-Controlled, Phase 1 Dose Escalation Trial to Evaluate the Safety and Immunogenicity of an Inactivated Chikungunya Virus Vaccine, HydroVax-005 CHIKV, in Healthy Adults. 2024.

76. A Phase I, Dose-escalation, Randomized, Single-blind, Placebo-controlled Trial to Evaluate the Safety, Reactogenicity, and Immunogenicity of PepGNP-ChikV, a Synthetic Nanoparticle-based T Cell Next-generational Vaccine Against Chikungunya in Healthy Adults. 2026.

77. August, A.; Attarwala, H.Z.; Himansu, S.; Kalidindi, S.; Lu, S.; Pajon, R.; Han, S.; Lecerf, J.M.; Tomassini, J.E.; Hard, M.; et al. A phase 1 trial of lipid-encapsulated mRNA encoding a monoclonal antibody with neutralizing activity against Chikungunya virus. *Nat Med* 2021, *27*, 2224-2233, doi:10.1038/s41591-021-01573-6.
