# Supplementary material for: Mapping Human Clinical Evidence for Chikungunya Vaccines: A Scoping Review of Immunogenicity, Durability, and Safety
Source: Vaccines (Basel). 2026 Jul 6;14(7):598. doi: 10.3390/vaccines14070598 (PMC13417312; doi:10.3390/vaccines14070598)
Supplement: Supplementary file 1 [file vaccines-14-00598-s001.zip › CHIKV_Supplementary_Table_S2_sw629.pdf]

Supplementary Table S2. Safety evidence summary of chikungunya vaccine candidates

Note. Records are cross-checked against the updated 77-reference library. Only records with extractable or contextually relevant human safety information are summarized. Registry/protocol-only records without results, superseded preliminary abstracts without unique safety information, preclinical-only records, news/commentary, and non-active-vaccine interventions are not expanded here. These safety summaries are product-specific and should not be interpreted as comparative safety estimates across vaccine platforms.

Abbreviations. AE = adverse event; AESI = adverse event of special interest; AR = adverse reaction; FU = follow-up; IM = intramuscular; MAAE = medically attended adverse event; NR = not reported; SAE = serious adverse event; VLP = virus-like particle.

TSI-GSD-218 (n=1)

Safety population	Safety follow-up and endpoints	Extractable safety findings	SAE/death and interpretation	Ref.
Phase II randomized placebo-controlled trial; n=73 healthy adults in the USA, aged 18–40 years; 59 vaccine and 14 placebo recipients.	Clinical checks during the first 28 days; laboratory safety at days 0, 14, and 28; serology follow-up to 1 year.	Local symptoms/signs were similar to placebo: 12/59 (20%) vaccine vs 3/14 (21%) placebo. Any systemic symptoms/signs: 34/59 (58%) vs 9/14 (64%). Transient isolated arthralgia occurred in 5/59 vaccinees and 0/14 placebo recipients; flu-like symptoms occurred in 13/59 vs 4/14.	No major safety imbalance was reported. Exact SAE/death counts were not extractable from the table source; no death signal was reported.	[1]

CHIKV VLP program: VRC-CHKVLP059; PXVX0317/Vimkunya (n=7)

Safety population	Safety follow-up and endpoints	Extractable safety findings	SAE/death and interpretation	Ref.
Phase I open-label dose-escalation trial; n=25 healthy adults in the USA, aged 18–50 years; VRC-CHKVLP059 three-dose regimen.	Solicited local/systemic reactogenicity for 7 days after each injection; AEs to 28 days after each vaccination; SAEs across study follow-up.	All injections were well tolerated. Solicited reactogenicity was mild only: 9/25 (36%) reported mild local reactogenicity and 10/25 (40%) reported mild systemic reactogenicity at least once. No moderate or severe reactogenicity was reported.	No serious adverse events were reported; no arthralgia was reported.	[2]
Phase II randomized placebo-controlled trial in endemic regions; n=400 adults aged 18–60 years; 201 vaccine and 199 placebo recipients.	Solicited local/systemic reactogenicity after two injections; unsolicited AEs through 28 days after the second injection; SAEs and chronic medical events through 72 weeks.	At least one local symptom occurred in 64 vaccine recipients (32%) vs 37 placebo recipients (19%). Systemic reactogenicity was reported by 87 vaccine recipients (44%) and was generally mild or moderate; systemic rates were not significantly different from placebo.	No vaccine-related SAE was reported. Sixteen potentially related mild/moderate unsolicited AEs occurred in 11 participants and resolved without sequelae. Sixteen unrelated SAEs occurred: 4 in the vaccine group and 12 in the placebo.	[3]
Phase II randomized trial of PXVX0317/Vimkunya formulations; n=415 adults aged 18–45 years in the USA.	Solicited local/systemic AEs for 7 days after each injection; unsolicited AEs to 28 days after last primary-series dose; SAEs to study completion, up to approximately 2 years in selected groups.	Injection-site pain was the most common solicited AE: 12/53 (23%) in the unadjuvanted group and 111/356 (31%) in adjuvanted groups. Solicited systemic events were mainly fatigue, headache, and myalgia; most were mild/moderate. Joint pain after the first vaccination was reported in 25/409 (6%) and resolved.	Twelve SAEs occurred in 9 participants; none were vaccine-related and all resolved. No vaccine-related SAE was reported.	[4]
Post hoc serostatus subgroup analysis of the VRC704 Phase II VLP trial; vaccine recipients included 39 baseline seropositive and 155 baseline seronegative adults.	Safety compared by baseline CHIKV-luc serostatus; solicited reactogenicity for 7 days after vaccination and unsolicited AEs to 28 days.	Solicited systemic AE rates were not meaningfully different by baseline serostatus. Injection-site swelling was more frequent in seropositive vs seronegative vaccine recipients (10.3% vs 0.6%) and was mild or moderate.	No vaccine-related SAE was reported in this subgroup analysis.	[5]
Phase II open-label study of PXVX0317/Vimkunya after prior alphavirus vaccination; n=60 adults aged 18–65 years in the USA, with 30 previous alphavirus vaccine recipients and 30 matched alphavirus-naive controls.	Solicited AEs for 7 days; unsolicited AEs to day 29; SAEs to day 182.	Solicited AEs were reported by 16/30 (53.3%) previous alphavirus vaccine recipients and 12/30 (40.0%) naive controls. Injection-site pain occurred in 13/30 (43.3%) vs 9/30 (30.0%). Common systemic events included headache and myalgia (each 11/60; 18.3%).	No vaccine-related SAE or potentially life-threatening AE was reported. One unrelated SAE occurred in a participant with a pre-existing metatarsal fracture exacerbation.	[6]
Phase III randomized placebo-controlled trial of Vimkunya; n=3254 dosed participants aged 12–64 years; vaccine n=2790, placebo n=464.	Solicited AEs to day 8; unsolicited AEs to day 29; AESIs, medically attended AEs, and SAEs to day 183.	Any AE: 1257/2790 (45.1%) vaccine vs 161/464 (34.7%) placebo. Local solicited AEs: 661/2765 (23.9%) vs 49/458 (10.7%); injection-site pain: 656/2764 (23.7%) vs 49/458 (10.7%). Systemic solicited AEs: 891/2765 (32.2%) vs 114/458 (24.9%); common events were fatigue, headache, and myalgia.	SAEs: 23/2790 (0.8%) vaccine vs 1/464 (0.2%) placebo. One SAE was initially assessed as possibly related by the site investigator but was considered unrelated by the sponsor and safety monitoring review. One fatal AE in the vaccine group was unrelated to treatment.	[7]
Phase III randomized placebo-controlled trial in adults aged ≥65 years; n=413 randomized; vaccine n=206, placebo n=207.	Solicited AEs to day 8; unsolicited AEs to day 29; AESIs, medically attended AEs, and SAEs to day 183.	Any AE: 47/206 (23%) vaccine vs 52/207 (25%) placebo. Local solicited AEs: 11/206 (5%) vs 4/207 (2%); injection-site pain: 11/206 (5%) vs 3/207 (1%). Systemic solicited AEs: 22/206 (11%) vs 27/207 (13%); most events were grade 1–2.	SAEs: 4/206 (2%) vaccine vs 3/207 (1%) placebo; none were treatment-related. One fatal SAE occurred in each group, and neither was treatment-related. No vaccine-related SAE or death occurred.	[8]

MV-CHIK / V184 (n=4)

Safety population	Safety follow-up and endpoints	Extractable safety findings	SAE/death and interpretation	Ref.
Phase I dose-escalation trial of MV-CHIK; n=42 healthy adults in Austria.	Safety and tolerability monitoring after MV-CHIK vaccination and booster schedules.	Overall safety profile was good. Adverse events increased with vaccine dose and injected volume; exact category rates were not fully extractable from the available abstract-level record.	No vaccination-related SAE was reported in the available record.	[9]
Conference abstract on MV-CHIK in participants with previous natural chikungunya exposure in Puerto Rico; exact sample details not fully extractable.	Solicited AEs after two IM doses and unsolicited AEs for 1 year were described at abstract level.	The abstract did not suggest higher solicited AE rates in pre-exposed participants. Reported pre-exposed vs unexposed rates included fatigue 31% vs 33%, malaise 13% vs 44%, headache 31% vs 72%, joint pain 31% vs 22%, and injection-site pain 19% vs 61%.	SAE/death details were not fully extractable from the abstract-level record. Retain as unique context for previous-exposure safety, but treat as lower-certainty evidence.	[10]
Phase II randomized trial in Austria and Germany; n=263 adults, including 229 MV-CHIK recipients and 34 control-vaccine recipients.	Solicited AEs collected after vaccination; unsolicited AEs and SAEs collected across study follow-up to day 224.	Solicited AEs were similar between MV-CHIK and control groups: 168/229 (73%) vs 24/34 (71%). Unsolicited AEs: 116/229 (51%) vs 17/34 (50%). Most solicited AEs were mild or moderate; severe AEs were not significantly more frequent in MV-CHIK groups.	Six participants had SAEs, four in MV-CHIK and two in control groups; all were unrelated. Seven arthritis-related AESIs occurred in MV-CHIK recipients; two were considered possibly related, and the between-group difference was not significant.	[11]

Safety population	Safety follow-up and endpoints	Extractable safety findings	SAE/death and interpretation	Ref.
Phase I double-blind placebo-controlled trial; n=180 adults across six cohorts; 25 MV-CHIK and 5 placebo recipients per cohort.	Solicited AEs through day 15 after each injection; unsolicited nonserious AEs through day 29 after each injection; SAEs through 6 months after last injection.	Solicited AEs were common but mostly mild/moderate. Among vaccine recipients, 83% had a solicited AE. High-dose recipients had more injection-site AEs than low-dose recipients (85% vs 59%). Common systemic AEs included headache, fatigue, and malaise.	Five SAEs in four participants occurred; none were related to study product. No vaccine recipient had an AESI; no prolonged vaccine-related arthralgia was observed.	[12]

VLA1553 / IXCHIQ (n=8)

Safety population	Safety follow-up and endpoints	Extractable safety findings	SAE/death and interpretation	Ref.
Phase I randomized dose-finding trial; n=120 adults aged 18–45 years in the USA; low, medium, and high dose groups.	Solicited injection-site and systemic reactions to day 14; unsolicited AEs to day 28; AESI and SAE monitoring through 12 months and revaccination follow-up.	Local reactogenicity was low: fewer than 7% of vaccinees reported a local AE. Solicited systemic reactions were dose-dependent: 11/31 (35.5%) low dose, 12/30 (40.0%) medium dose, and 40/59 (67.8%) high dose. Common systemic events included fever, headache, fatigue, and muscle pain.	No AESI and no vaccine-related SAE were reported. One unrelated SAE of polytrauma after a car accident occurred.	[13]
Pivotal Phase III randomized placebo-controlled trial; n=4115 safety population, including 3082 VLA1553 and 1033 placebo recipients aged ≥18 years.	Solicited injection-site and systemic AEs for 10 days; unsolicited AEs to 28 days; AESIs days 2–21; all AEs and SAEs to day 180.	Any AE: 1926/3082 (62.5%) VLA1553 vs 463/1033 (44.8%) placebo. Solicited injection-site AEs: 463/3082 (15.0%). Solicited systemic AEs: 1547/3082 (50.2%) vs 278/1033 (26.9%). Common AEs included headache, fatigue, myalgia, arthralgia, injection-site pain, and pyrexia.	SAEs: 46/3082 (1.5%) vs 8/1033 (0.8%). Two related SAEs occurred in VLA1553 recipients (mild myalgia and SIADH/hyponatremia), both recovered. AESIs: 10/3082 (0.3%) vs 1/1033 (0.1%).	[14]
Pooled safety analysis of VLA1553 across Phase I, pivotal Phase III, and lot-consistency studies; VLA1553 n=3520, placebo n=1033, adults aged ≥18 years.	Pooled solicited injection-site/systemic AEs (10–14 days), unsolicited AEs to days 29 and 180, AESIs to day 28, medically attended AEs, SAEs to day 180, and pregnancies.	Overall AEs occurred in 63.7% of VLA1553 recipients vs 44.7% placebo. Solicited injection-site AEs: 15.5% vs 11.1%; solicited systemic AEs: 50.9% vs 26.9%; unsolicited day-29 AEs: 22.7% vs 13.4%. Arthralgia occurred in 16.7% vs 4.8%, with none medically attended.	MAAEs: 12.4% vs 11.3%; AESIs: 0.3% vs 0.1%; SAEs: 1.5% vs 0.8%. Two VLA1553-related SAEs resolved without sequelae. Three deaths occurred, none vaccine-related; no vaccine-related adverse pregnancy outcomes were reported.	[15]
Phase III lot-to-lot consistency study; n=408 vaccinated adults aged 18–45 years in the safety population.	Solicited and unsolicited AEs through 6 months, with comparison across three manufacturing lots.	AEs occurred in 72.5% of participants, without significant lot differences. Related solicited systemic AEs occurred in 53.9% and related solicited local AEs in 19.4%. AEs were mostly mild/moderate and usually resolved within 3 days.	Severe AEs occurred in 3.9% of participants, with 2.7% classified as related. Six SAEs were reported and none were related to VLA1553.	[16]
Phase 3b single-arm persistence/safety follow-up; n=363 adults previously vaccinated with VLA1553 in the pivotal Phase III trial.	Safety focus from 6 months to 2 years after vaccination: ongoing AESIs at transition and new SAEs during follow-up.	No AESI was ongoing at the 6-month transition into the follow-up study.	Ten SAEs occurred in nine participants between 6 months and 2 years, including one death due to drug overdose; all were determined unrelated to VLA1553. No long-term vaccine-related SAE was identified.	[17]
Final 12-month Phase III adolescent trial in Brazil; n=754 vaccinated adolescents aged 12–17 years; VLA1553 n=502, placebo n=252.	Solicited AEs to day 10; unsolicited AEs to 6 months; SAEs, AESIs, and recurring arthralgia monitoring to 12 months.	Related AEs were more frequent with VLA1553 than placebo: 352/502 (70.1%) vs 122/252 (48.4%). Common related AEs included headache, injection-site pain, myalgia, fever, and fatigue. Early-onset AESIs occurred in 17/502 (3.4%) vs 2/252 (0.8%).	One possibly related SAE of high-grade fever occurred. No deaths occurred. Nine adolescents had recurring arthralgia episodes, usually mild and short-lived; one long-term polyarthralgia case was classified unrelated to VLA1553.	[18]
Post-authorization safety review of IXCHIQ/VLA1553 during early real-world use, including the 2024–2025 La Reunion outbreak; approximately 55,900 doses administered worldwide by 31 Aug 2025.	Serious adverse events reported from 9 Nov 2023 to 31 Aug 2025 across major pharmacovigilance systems and manufacturer reports.	Thirty-five SAEs were reported, corresponding to 6.3 per 10,000 doses. Adults aged ≥65 years represented 33% of doses but 77% of SAEs. Median SAE age was 73 years and 91% had comorbidities. Events included systemic chikungunya-like reactions (15), neurological events (13), cardiac events (3), and renal events (3).	Three fatalities occurred in older men with multimorbidity. One vaccine-strain encephalitis case was considered probably related. No batch/manufacturing issue was identified; regulatory reviews concluded that benefit-risk remained favorable within authorized and nationally recommended populations.	[19]
Phase II observer-blind pediatric dose–response trial in Dominican Republic and Honduras; n=304 children aged 1–11 years; half-dose n=119, full-dose n=124, Nimenrix control n=61.	Solicited AEs to day 14; unsolicited AEs, medically attended AEs, SAEs, and AESIs through day 29 in prespecified interim analysis.	Solicited injection-site AEs were mainly tenderness (10.9%) and pain (8.6%). Solicited systemic AEs were mainly fever (12.5%) and headache (11.5%; headache not solicited in ages 1–2 years). Unsolicited AEs were mostly infections and infestations (10.0%–20.5%).	The most common medically attended AE was fever (2.6%). Three AESIs were reported, all in the half-dose group; none were serious and all resolved within 1 week. No deaths were reported through day 29.	[20]

ChAdOx1 Chik (n=1)

Safety population	Safety follow-up and endpoints	Extractable safety findings	SAE/death and interpretation	Ref.
Phase I first-in-human dose-escalation trial; n=24 healthy adults aged 18–50 years in the UK.	Solicited local/systemic AEs for 7 days; unsolicited AEs to day 28; SAEs across study duration.	ChAdOx1 Chik was reported as well tolerated at doses up to 5 x 10^10 viral particles. A total of 112 solicited local/systemic AEs occurred; most were mild (79/112; 70.5%) or moderate (27/112; 24.1%) and all resolved within 7 days. Injection-site pain was the most common local AE (79.2%). Fatigue was the most common systemic AE.	One SAE was reported and was deemed unrelated. No serious adverse reaction to ChAdOx1 Chik was reported.	[21]

mRNA-1388 / VAL-181388 (n=1)

Safety population	Safety follow-up and endpoints	Extractable safety findings	SAE/death and interpretation	Ref.
Phase I randomized placebo-controlled dose-ranging trial; n=60 healthy adults aged 18–49 years in a CHIKV-nonendemic region.	Solicited local/systemic adverse reactions for 7 days after each dose; unsolicited AEs to 28 days after each dose; SAEs and AESIs monitored.	Solicited local ARs occurred in 41/59 (69.5%) after dose 1 and 32/57 (56.1%) after dose 2. Solicited systemic ARs occurred in 16/59 (27.1%) after dose 1 and 21/57 (36.8%) after dose 2. Common local ARs were pain and tenderness; common systemic ARs were headache, generalized myalgia, and fatigue.	No deaths or AESIs were reported. One treatment-related SAE occurred after dose 2: grade 4 aspartate aminotransferase increase. One grade 3 neutropenia led to withdrawal after dose 1 and was deemed unrelated.	[22]

Records excluded from Table S2: registry/protocol-only records without results, superseded preliminary abstracts without unique safety information, preclinical-only records, news/commentary records, and non-active-vaccine interventions.

1. Edelman, R.; Tacket, C.O.; Wasserman, S.S.; Bodison, S.A.; Perry, J.G.; Mangiafico, J.A. Phase II safety and immunogenicity study of live chikungunya virus vaccine TSI-GSD-218. *American Journal of Tropical Medicine and Hygiene* 2000, *62*, 681-685, doi:10.4269/ajtmh.2000.62.681.
2. Chang, L.J.; Dowd, K.A.; Mendoza, F.H.; Saunders, J.G.; Sitar, S.; Plummer, S.H.; Yamshchikov, G.; Sarwar, U.N.; Hu, Z.; Enama, M.E.; et al. Safety and tolerability of chikungunya virus-like particle vaccine in healthy adults: A phase 1 dose-escalation trial. *The Lancet* 2014, *384*, 2046-2052, doi:10.1016/S0140-6736(14)61185-5.
3. Chen, G.L.; Coates, E.E.; Plummer, S.H.; Carter, C.A.; Berkowitz, N.; Conan-Cibotti, M.; Cox, J.H.; Beck, A.; O'Callahan, M.; Andrews, C.; et al. Effect of a Chikungunya Virus-Like Particle Vaccine on Safety and Tolerability Outcomes A Randomized Clinical Trial. *Jama-Journal of the American Medical Association* 2020, *323*, 1369-1377, doi:10.1001/jama.2020.2477.
4. Bennett, S.R.; McCarty, J.M.; Ramanathan, R.; Mendy, J.; Richardson, J.S.; Smith, J.; Alexander, J.; Ledgerwood, J.E.; de Lame, P.A.; Royalty Tredo, S.; et al. Safety and immunogenicity of PXVX0317, an aluminium hydroxide-adjuvanted chikungunya virus-like particle vaccine: a randomised, double-blind, parallel-group, phase 2 trial. *The Lancet Infectious Diseases* 2022, *22*, 1343-1355, doi:10.1016/S1473-3099(22)00226-2.
5. McCarty, J.M.; Bedell, L.; Mendy, J.; Coates, E.E.; Chen, G.L.; Ledgerwood, J.E.; Tredo, S.R.; Warfield, K.L.; Richardson, J.S. Chikungunya virus virus-like particle vaccine is well tolerated and immunogenic in chikungunya seropositive individuals. *Vaccine* 2023, *41*, 6146-6149, doi:10.1016/j.vaccine.2023.08.086.
6. Hamer, M.J.; McCarty, J.M.; Pierson, B.C.; Regules, J.A.; Mendy, J.; Sanborn, A.; Gardner, C.L.; Haller, J.M.; Gregory, M.K.; Liggett, D.L.; et al. Safety and immunogenicity of an adjuvanted chikungunya virus virus-like particle (CHIKV VLP) vaccine in previous recipients of other alphavirus vaccines versus alphavirus vaccine-naive controls: an open-label, parallel-group, age-matched, sex-matched, phase 2 randomised controlled study. *Lancet Microbe* 2025, *6*, doi:10.1016/j.lanmic.2024.101000.
7. Richardson, J.S.; Anderson, D.M.; Mendy, J.; Tindale, L.C.; Muhammad, S.; Loreth, T.; Tredo, S.R.; Warfield, K.L.; Ramanathan, R.; Caso, J.; et al. Chikungunya virus virus-like particle vaccine safety and immunogenicity in adolescents and adults in the USA : a phase 3, randomised, double-blind, placebo-controlled trial. *Lancet* 2025, *405*, 1343-1352, doi:10.1016/s0140-6736(25)00345-9.
8. Tindale, L.C.; Richardson, J.S.; Anderson, D.M.; Mendy, J.; Muhammad, S.; Loreth, T.; Tredo, S.R.; Ramanathan, R.; Jenkins, V.A.; Bedell, L.; et al. Chikungunya virus virus-like particle vaccine safety and immunogenicity in adults older than 65 years: a phase randomised, double-blind, placebo-controlled trial. *Lancet* 2025, *405*, 1353-1361, doi:10.1016/s0140-6736(25)00372-1.
9. Ramsauer, K.; Schwameis, M.; Firbas, C.; Müllner, M.; Putnak, R.J.; Thomas, S.J.; Desprès, P.; Tauber, E.; Jilma, B.; Tangy, F. Immunogenicity, safety, and tolerability of a recombinant measles-virus-based chikungunya vaccine: A randomised, double-blind, placebo-controlled, active-comparator, first-in-man trial. *The Lancet Infectious Diseases* 2015, *15*, 519-527, doi:10.1016/S1473-3099(15)70043-5.
10. Ramsauer, K.; Reisinger, E.; Firbas, C.; Wiedermann-Schmidt, U.; Beubler, E.; Pfeiffer, A.; Müllner, M.; Aberle, J.; Tauber, E. Phase 2 clinical results: Chikungunya vaccine based on measles vector (MV-CHIK) induces humoral and cellular responses in the presence of pre-existing anti measles immunity. *International Journal of Infectious Diseases* 2019, *79*, 118, doi:10.1016/j.ijid.2018.11.291.
11. Reisinger, E.C.; Tschismarov, R.; Beubler, E.; Wiedermann, U.; Firbas, C.; Loebermann, M.; Pfeiffer, A.; Muellner, M.; Tauber, E.; Ramsauer, K. Immunogenicity, safety, and tolerability of the measles-vectored chikungunya virus vaccine MV-CHIK: a double-blind, randomised, placebo-controlled and active-controlled phase 2 trial. *Lancet* 2019, *392*, 2718-2727, doi:10.1016/s0140-6736(18)32488-7.
12. Winokur, P.; Hegmann, T.E.; El Sahly, H.M.; Anderson, E.J.; Grp, D.S. A Phase 1 Double-Blinded Trial to Evaluate Safety, Immunogenicity, and Dosing of Measles-Vectored Chikungunya Virus Vaccine (MV-CHIK) in Healthy Adults. *Journal of Infectious Diseases* 2026, *233*, e641-e645, doi:10.1093/infdis/jiaf571.
13. Wressnigg, N.; Hochreiter, R.; Zoihsl, O.; Fritzer, A.; Bézay, N.; Klingler, A.; Lingnau, K.; Schneider, M.; Lundberg, U.; Meinke, A.; et al. Single-shot live-attenuated chikungunya vaccine in healthy adults: a phase 1, randomised controlled trial. *The Lancet Infectious Diseases* 2020, *20*, 1193-1203, doi:10.1016/S1473-3099(20)30238-3.
14. Schneider, M.; Narciso-Abraham, M.; Hadl, S.; McMahon, R.; Toepfer, S.; Fuchs, U.; Hochreiter, R.; Bitzer, A.; Kosulin, K.; Larcher-Senn, J.; et al. Safety and immunogenicity of a single-shot live-attenuated chikungunya vaccine: a double-blind, multicentre, randomised, placebo-controlled, phase 3 trial. *The Lancet* 2023, *401*, 2138-2147, doi:10.1016/S0140-6736(23)00641-4.
15. Maurer, G.; Buerger, V.; Larcher-Senn, J.; Erlsbacher, F.; Dubischar, K.; Eder-Lingelbach, S.; Jaramillo, J.C. Pooled safety evaluation for a new single-shot live-attenuated chikungunya vaccine. *Journal of Travel Medicine* 2024, *31*, doi:10.1093/jtm/taae133.
16. McMahon, R.; Fuchs, U.; Schneider, M.; Hadl, S.; Hochreiter, R.; Bitzer, A.; Kosulin, K.; Koren, M.; Mader, R.; Zoihsl, O.; et al. A randomized, double-blinded Phase 3 study to demonstrate lot-to-lot consistency and to confirm immunogenicity and safety of the live-attenuated chikungunya virus vaccine candidate VLA1553 in healthy adults. *Journal of Travel Medicine* 2024, *31*, doi:10.1093/jtm/taad156.
17. McMahon, R.; Toepfer, S.; Sattler, N.; Schneider, M.; Narciso-Abraham, M.; Hadl, S.; Hochreiter, R.; Kosulin, K.; Mader, R.; Zoihsl, O.; et al. Antibody persistence and safety of a live-attenuated chikungunya virus vaccine up to 2 years after single-dose administration in adults in the USA: a single-arm multicentre, phase 3b study. *Lancet Infectious Diseases* 2024, *24*, 1383-1392, doi:10.1016/s1473-3099(24)00357-8.
18. Buerger, V.; Pfeiffer, A.; Schoengrundner, P.; Seebacher, J.; Hochreiter, R.; Kosulin, K.; Zoihsl, O.; Weisova, P.; Mader, R.; Loch, A.P.; et al. Safety and immunogenicity of a live-attenuated chikungunya virus vaccine in adolescents: final results from a 12-month, double-blind, randomised, placebo-controlled, phase 3 trial in endemic areas of Brazil. *Lancet Infect Dis* 2026, *26*, 417-428, doi:10.1016/s1473-3099(25)00631-0.
19. Vondeling, G.T.; Croda, J.; Jelinek, T.; Kassianos, G.; Kollaritsch, H.; Nguyen, L.B.L.; Ribeiro, G.S.; Schmidt-Chanasit, J.; Thomas, S.J.; Unger, Z.; et al. Post-marketing safety evaluation of the live-attenuated chikungunya vaccine (IXCHIQ). *Vaccine* 2026, *79*, doi:10.1016/j.vaccine.2026.128491.
20. Weisová, P.; Scheiblauer, S.; Ecker, J.; Schneider, M.; Hochreiter, R.; Bitzer, A.; Kosulin, K.; Schoengrundner, P.; Fuchs, U.; Rodeles, L.; et al. Live-attenuated chikungunya vaccine in children: a randomized phase 2 trial. *Nature Medicine* 2026, *32*, 561-571, doi:10.1038/s41591-025-04197-2.
21. Folegatti, P.M.; Harrison, K.; Preciado-Llanes, L.; Lopez, F.R.; Bittaye, M.; Kim, Y.C.; Flaxman, A.; Bellamy, D.; Makinson, R.; Sheridan, J.; et al. A single dose of ChAdOx1 Chik vaccine induces neutralizing antibodies against four chikungunya virus lineages in a phase 1 clinical trial. *Nature Communications* 2021, *12*, doi:10.1038/s41467-021-24906-y.
22. Shaw, C.A.; August, A.; Bart, S.; Booth, P.G.J.; Knightly, C.; Brasel, T.; Weaver, S.C.; Zhou, H.; Panther, L. A phase 1, randomized, placebo-controlled, dose-ranging study to evaluate the safety and immunogenicity of an mRNA-based chikungunya virus vaccine in healthy adults. *Vaccine* 2023, *41*, 3898-3906, doi:10.1016/j.vaccine.2023.04.064.
